# Imputation-Based Whole-Genome Sequence Association Study Reveals Constant and Novel Loci for Hematological Traits in a Large-Scale Swine F_2_ Resource Population

**DOI:** 10.3389/fgene.2018.00401

**Published:** 2018-10-22

**Authors:** Guorong Yan, Tianfu Guo, Shijun Xiao, Feng Zhang, Wenshui Xin, Tao Huang, Wenwu Xu, Yiping Li, Zhiyan Zhang, Lusheng Huang

**Affiliations:** State Key Laboratory for Pig Genetic Improvement and Production Technology, Jiangxi Agricultural University, Nanchang, China

**Keywords:** GWAS, imputation, hematology, pig, whole-genome sequence

## Abstract

The whole-genome sequences of progenies with low-density single-nucleotide polymorphism (SNP) genotypes can be imputed with high accuracy based on the deep-coverage sequences of key ancestors. With this imputation technology, a more powerful genome-wide association study (GWAS) can be carried out using imputed whole-genome variants and the phenotypes of interest to overcome the shortcomings of low-power detection and the large confidence interval derived from low-density SNP markers in classic association studies. In this study, 19 ancestors of a large-scale swine F_2_ White Duroc × Erhualian population were deeply sequenced for their genome with an average coverage of 25×. Considering 98 pigs from 10 different breeds with high-quality deep sequenced genomes, we imputed the whole genomic variants of 1020 F_2_ pigs genotyped by the PorcineSNP60 BeadChip with high accuracy and obtained 14,851,440 sequence variants after quality control. Based on this, 87 novel quantitative traits loci (QTLs) for 18 hematological traits at three different physiological stages of the F_2_ pigs were identified, among which most of the novel QTLs have been repeated in two of the three stages. Literature mining pinpointed that the *FGF14* and *LCLAT1* genes at SSC11 and SSC3 may affect the MCH at day 240 and MCV at day 18, respectively. The present study shows that combining high-quality imputed genomic variants and correlated phenomic traits into GWAS can improve the capability to detect QTL considerably. The large number of different QTLs for hematological traits identified at multiple growth stages implies the complexity and time specificity of these traits.

## Introduction

Hematological traits, which are associated with the immune system (Oddgeirsson et al., 1988; Muller and Brem, 1991), have been widely used as biomarkers to detect the signs of diseases and disease severity in human and animals. Hematological cells consist of three different kinds of cells: leukocytes (white blood cells, WBCs), erythrocytes (red blood cells, RBCs), and platelets, which perform different roles in blood (Tullis, 1952). WBCs are called immune cells and play the function of defending against bacteria or fungi and protecting an organism from disease. RBCs exchange oxygen and carbon dioxide in the respiration of vertebrate animals. Platelets play key roles in wound healing and inflammatory response (Toby Simon, 2001). Identifying highly associated SNPs and deciphering the genetic mechanism of hematological traits would help in dealing with hematological diseases.

The researchers have paid considerable attention to hematological traits in humans. The heritabilities of RBC, WBC, and platelet count have been estimated to be 0.42, 0.62, and 0.57, respectively, indicating moderately or highly heritable traits (Garner et al., 2000). Some heritability studies on twins have proven that hematological traits are highly heritable and tightly interactive (Evans et al., 1999; Garner et al., 2000). In 2012, common genetic factors for hematological traits in humans were reviewed in detail by Okada and Kamatani (2012). In the last 5 years, several missense variants in *CXCR2* associated with the reduction of WBC count, a common missense variant in *CPS1* and a rare synonymous variant in *GFI1B* that caused a lower platelet count, and a mass of genes and loci-related hematological parameters have been detected by adopting the genome-wide association studies (GWAS) approach (van der Harst et al., 2012; Auer et al., 2014; Polfus et al., 2016; Ulirsch et al., 2016). Due to the strong similarity with human physiological characteristics, the domestic pig serves as a suitable model animal in elucidating the genetic mechanism of hematological traits in humans (Swindle et al., 2012).

To date, a total of 356 quantitative trait loci (QTLs) associated with swine hematological traits have been identified by linkage mapping recorded in the AnimalQTLdb database (Hu et al., 2007), but because of the large confidence intervals and large number of genes in these QTL regions, the identification of potential causal genes would be obstructed to some extent. With the discovery of massive numbers of genetic markers and the advent of high-throughput technology to genotype animals for hundreds of thousands of single-nucleotide polymorphisms (SNPs) in a cost-effective way, GWAS is widely applied in the fields of human and livestock for traits of interest. For the hematological traits in pigs, hundreds of significant SNPs located in the whole genome except chromosomes 14 and 16 were identified using the PorcineSNP60 BeadChip (Illumina Inc., San Diego, CA, United States) based on GWAS in diversified pig populations (Luo et al., 2012; Wang et al., 2013; Zhang et al., 2013, 2014; Jung et al., 2014; Ponsuksili et al., 2016). The effects of uncovered significant loci are population- or breed-specific and only a small fraction of loci are consistent among these studies, implying the complexity and heterogeneity of hematological traits. On the other hand, those massive significant SNPs can explain only a relatively small fraction of phenotypic variance, ranging from 4.75 to 19.41% of hematological traits (Zhang et al., 2014). The remaining and missing heritability has been discussed in 2009 (Manolio et al., 2009). A fairly stringent statistical threshold, numerous small-effect loci affecting traits, and the relative low linkage disequilibrium (LD) among markers should be most likely responsible for the missing heritability. All these genes with small effects can together explain the majority of the genetic variance for most of these traits (Yang et al., 2010).

In our previous study, we performed a GWAS analysis in a White Duroc × Erhualian F_2_ resource population using the PorcineSNP60 BeadChip, and we identified 185 significant SNPs distributed on chromosomes 1, 4, 5, 7, 8, 10, 11, 12, 13, 17, and 18, affecting hematological traits at three different growth stages (Zhang et al., 2013). To obtain the majority of the remaining genetic variance and to overcome the weakness of low efficiency caused by low LD in detecting small-effect QTLs when using low-density BeadChip (Supplementary Figure S1), we carried out a single- and multitrait association study using imputation-based whole-genome sequence data in the same F_2_ resource population. As causal variants and higher LD may have been included in the whole-genome sequences, the detection power for small-effect variants would improve. We herein aim to explore more loci associated with hematological traits using a whole-genome sequence based on imputation in pigs.

## Materials and Methods

### Ethics Statement

All the processes involving animals are in accordance with the care and the guidelines of experimental animals established by the Ministry of Agriculture and Rural Affairs of the China. The ethics committee of Jiangxi Agricultural University specifically approved this study.

### Animals and Phenotypic Measurements

The description of the White Duroc × Erhualian F_2_ resource population and the related method of phenotypic measurement have been described in detail in our previous publications (Zou et al., 2008; Guo et al., 2009; Yang et al., 2009). In short, a total of 1912 F_2_ individuals were produced from two White Duroc boars and seventeen Erhualian sows. All F_2_ piglets were fed under the same circumstance in a pig farm at Jiangxi Agricultural University. Eighteen hematological parameters from 1449 F_2_ individuals were measured at three growth-age stages, i.e., days at 18, 46, and 240. These hematological parameters include seven erythroid parameters [hematocrit (HCT), hemoglobin (HGB), mean corpuscular hemoglobin (MCH), mean corpuscular hemoglobin concentration (MCHC), mean corpuscular volume (MCV), red blood cell count (RBC), and red blood cell volume distribution width (RDW)], seven leukocyte parameters [granulocyte count (GRAN), granulocyte count percentage (GRAR), monocyte count (MON), monocyte count percentage (MONR), lymphocyte count (LYM), lymphocyte count percentage (LYMA), and white blood cell count (WBC)], and four platelet parameters [plateletcrit (PCT), platelet distribution width (PDW), platelet count (PLT), and mean platelet volume (MPV)].

### Processing With Reference and Target Panels

One hundred and seventeen individuals with whole-genome sequence data produced by the Illumina HiSeq 2000 platform with an average depth of ∼25 coverages were introduced as the reference panel; of the individuals, 19 F_0_ individuals are the progenitors of the 933 F_2_ resource populations (target panel) and 98 unrelated pigs are from nine diverse breeds and one wild boar population. Sixty-nine unrelated pigs were used in a study of adaptation and introgression project (Ai et al., 2015). Briefly, these 69 pigs comprising 6 Bamaxiang, 6 Luchuan, 6 Wuzhishan, 6 Jiangxi Wild boar, 5 Erhualian, 6 Hetao, 6 Laiwu, 6 Min, 4 Gansu Tibetan, 6 Sichuan Tibetan, 6 Tibet Tibetan, and 6 Yunnan Tibetan represent both low- and high-latitude populations of pigs in China. After being sequenced, the raw reads were firstly trimmed based on a quality score threshold >15; reads that passed the chastity filtering were then aligned to the reference porcine genome assembly *Sus-scrofa* 10.2 (Groenen et al., 2012) using the Burrows–Wheeler aligner tool (Li and Durbin, 2009). Variants were called following the genome analysis toolkit (GATK) (McKenna et al., 2010) best practice protocol. PCR duplications were firstly marked by Picard MarkDuplicates^1^, and local realignments were performed with the GATK IndelRealigner option. Then, variants were filtered with the GATK VariantFiltration option, and insertions and deletions were performed with VCFtools (Danecek et al., 2011).

A subset of this resource population with 62,163 loci detected by PorcineSNP60 BeadChip was treated as the target panel. The target panel included 19 F_0_, 68 F_1_ and 933 F_2_. The 19 F_0_ ancestors included both resequencing variants and 60K genotypes. Individuals were genotyped by the PorcineSNP60 BeadChip following a standard protocol on an iScan system. A more complete description, including methods and the criteria of quality control (QC) of this SNP dataset can be found in our previous report (Zhang et al., 2013). To keep the alleles consistent with the reference panel, the following analysis was carried out. The information of forward or reverse strands of each SNP in the PorcineSNP60 BeadChip was firstly annotated by BLAST (Altschul et al., 1990). Then, the reversed SNPs in the target panel were flipped by PLINK (v1.9) software (Chang et al., 2015). The SNPs without specific information of chromosomes and (or) positions were excluded from further analysis.

### Haplotype Construction of Reference Panel and Target Panel

The accuracy of the haplotypes of the ancestors (F_0_) is critical for high-quality imputation of the F_2_ population. The number of individuals present in a population is a crucial factor in determining how well the phase can be estimated for haplotype construction. Browning and Browning (2011) and Williams et al. (2012) have fully demonstrated the relationship between sample size and haplotype accuracy; the more individuals, the better the haplotype estimation. In our study, to obtain more accurate phases of the 19 founders, an additional 98 unrelated pigs were added into the reference panel. The haplotypes of the reference panel were constructed by SHAPEIT (v2 r837) (Delaneau et al., 2013). To improve the accuracy of the construction of haplotypes, phase informative reads (PIRs) that span at least two heterozygous sites were firstly extracted from aligned *bam* files by using the *extractPIRs* program from SHAPEIT appendix, setting the *–base-quality* and *–read-quality* options to 20 and 30, respectively. Combined with the variants files, PIRs were then involved in haplotype construction using the standard Markov chain Monte Carlo iterative procedure of the phasing module (*-assemble*) in SHAPEIT and the window was set to 0.2 Mb by the *–window* parameter. Haplotypes of the target panel were constructed and improved by taking advantage of both linkage and LD information using DualPhase software; It firstly phased a large proportion of loci and individuals based on Mendelian rules and linkage information using the pedigree relationship of the F_2_ population; then, the remaining small part of the unphased loci were haplotyped based on a hidden Markov model (Druet and Georges, 2010).

### Imputation of Sequence Variants

Subsequently, the genotype imputation between the target and reference panels with known phase information were performed by IMPUTE2 (v.2.3.2) (Howie et al., 2009). Specifically, the option of *-use_prehased_g* was required to set IMPUTE2 in the prephasing mode. Then, the size of the region to be imputed on the current chromosome was set to a 5 Mb window with the *-int* option. Finally, the other options used in the imputation follow the default setting. Imputation accuracy was automatically assessed by an internal cross-validation strategy in IMPUTE2. Briefly, it masked the variants of one SNP in the target panel at a time, and then the masked genotypes were imputed by the information of the reference panel and the nearby studied variants. Then, the genotypic concordance rate and the squared correlation (*R^2^*) between the best-guess imputed and the original variants were calculated as imputation accuracy.

To investigate the imputation accuracy further, we carried out whole-genome resequencing for five experimental animals in the present study population including two F_1_ and three F_2_ individuals. The SNP calling of these five individuals was performed following the pipeline described above. The *R^2^* value between the sequenced variants and the best-guess imputed variants across chromosomes was calculated.

Furthermore, to obtain relatively accurate imputation results that would be used for the following GWAS, a series of methods were applied into the GEN file format from the imputation result. As the GEN file contains the information of genotype probability, we used a cutoff of 0.5 to convert the GEN file into a common PED file by GTOOL^2^ as it best balanced the imputation accuracy and the missing proportion in the next analysis process. Then, variants with a call rate <90% and a minor allele frequency (MAF) <0.01 were further excluded by PLINK.

### Phenotypic and Genetic Correlation

The estimated breeding value (EBV) of all experimental animals for 54 hematological traits were estimated by Bayesian sparse linear mixed models implemented in Gemma (v.0.94) (Zhou et al., 2013). The phenotypic correlation and the genetic correlation were calculated by using the *cor* function using the Pearson correlation coefficient in the stats package.

### Single-Trait GWAS Analysis

A univariate linear mixed model (see Eq. 1) implemented in GEMMA (v.0.94) is employed for the single-marker association test between variants and phenotypes (Zhou and Stephens, 2012), and is described in the following equation:

(1) $$\begin{array}{l} \text{y}=\text{Wα+xβ}+u+\text{ε};\;u\sim {\text{MVN}}_{n}(0,\;{\text{λτ}}^{-1}\text{K}),\\ \text{ ε}\sim {\text{MVN}}_{n}(0,\;{\text{τ}}^{-1}I_{n}) \end{array}$$

where **y** is the vector of phenotypic observation; W is a design matrix of covariates, including a column of 1 s; ***α*** is a vector of fixed effects (e.g., gender); x is a vector of genotypes at each locus; ***β*** is the effect of the marker; u is a vector of random effects following the multivariate normal distribution (see Eq. 1), in which *τ*^-1^ is the variance of the residual errors, λ is the ratio between *τ*^-1^ and the variance of polygenetic effects, and K is a kinship matrix estimated from whole-genome sequence variants; **𝜀** is a vector of errors following the multivariate normal distribution (see Eq. 1) and I*_n_* is an identity matrix. Using naïve Bonferroni corrections of 0.05 divided by the number of examined SNPs to correct multiple comparisons would lead to an overly conservative threshold in our study because these SNPs were highly correlated with each other. Pe’er et al. (2008) and Johnson et al. (2010) suggested that 5E-08 could serve as a genome-wide significant threshold in human GWASs based on independent haplotype blocks of an African population structure. Based on the assumption that an equal number of independent haplotype segments between pigs and humans are held, we used the same genome-wide threshold in our study.

### Multitrait GWAS Analysis

Hierarchical clustering of the traits in multitrait-GWAS was performed by using the function *corrplot* in the corrplot R package with the agglomeration method of “complete,” which employs the longest distance principle to generate clusters. Based on the clusters of phenotypic and genetic correlation from different stages, the multitrait association tests between variants and multiple correlated phenotypes were carried out by the multivariate linear mixed model (see Eq. 2) implemented in GEMMA (v. 0.94) (Zhou and Stephens, 2014), which is described in the following equation:

(2) $$\begin{array}{l} \text{Y}=\text{WA}+{\text{xβ}}^{T}+\text{U}+\text{E};\text{ U}\sim {\text{MVN}}_{n\times d}(0,\;{\text{K, V}}_{g}),\\ \text{ E}\sim {\text{MN}}_{n\times d}(0,\;{\text{I}}_{n\times n}{\text{, V}}_{e}) \end{array}$$

where *n* and *d* are the numbers of individuals and phenotypes, respectively. Y is a *n* by *d* matrix of phenotypic observation; W is an *n* by *c* design matrix of covariates, including a column of 1 s; A is a *c* by *d* matrix of fixed effects (e.g., gender); x is a vector of genotypes at each locus; ***β*** is a *d* vector of marker effect sizes for *d* phenotypes; U is an *n* by *d* matrix of random effects following the n × d matrix normal distribution (see Eq. 2), in which K is a relatedness matrix generated by genotypes of *n* individuals, and V*_g_* is a d by d matrix of genetic variance components; E is an *n* by *d* matrix of errors, in which I*_n×n_* is an *n* by *n* identity matrix, and V*_e_* is a d by d matrix of environmental variance components. The genome-wide significant threshold in multitrait GWAS was also set to 5E-08 as mentioned above.

## Results

### Phenotypic and Genetic Correlations

In this study, 1020 individuals in three generations, including 19 F_0_, 68 F_1_, and 933 F_2_ were genotyped by the PorcineSNP60 BeadChip. The number of individuals measured for each trait ranged from 181 to 924 (Supplementary Table S1). The detailed descriptive statistics of these 18 hematological traits were presented in our previous report (Zhang et al., 2013).

The detailed information about phenotypic correlation and genetic correlation across the three different stages were presented (Supplementary Figure S2). Some traits were excluded because there were too few records in those traits (Supplementary Table S1). There is a highly similar correlation pattern between the phenotypic correlation and genetic correlation for these traits; for example, traits with a high positive phenotypic correlation also have a high positive genetic correlation and vice versa. To further explore potential common genetic factors for multiple traits, we performed clustering analysis for the hematological traits using their genetic correlation matrix. Briefly, HCT, HGB, MCH, and MCV at day 18 were clustered to one group (Supplementary Figure S2A). At day 46, there was also only one cluster with many significant positive correlations among traits including GRAN, GRAR, MCH, and MCHC (Supplementary Figure S2B). For day 240, RDW, PCT, PLT, LYM, and LYMA were clustered to one group and MON, MONR, GRAN, GRAR, RBC, HCT, and HGB traits were clustered into another group (Supplementary Figure S2C). Here, a correlation cluster including at least four traits was used in the multitrait GWAS analysis process.

### Summary Imputation

**FIGURE 1:**
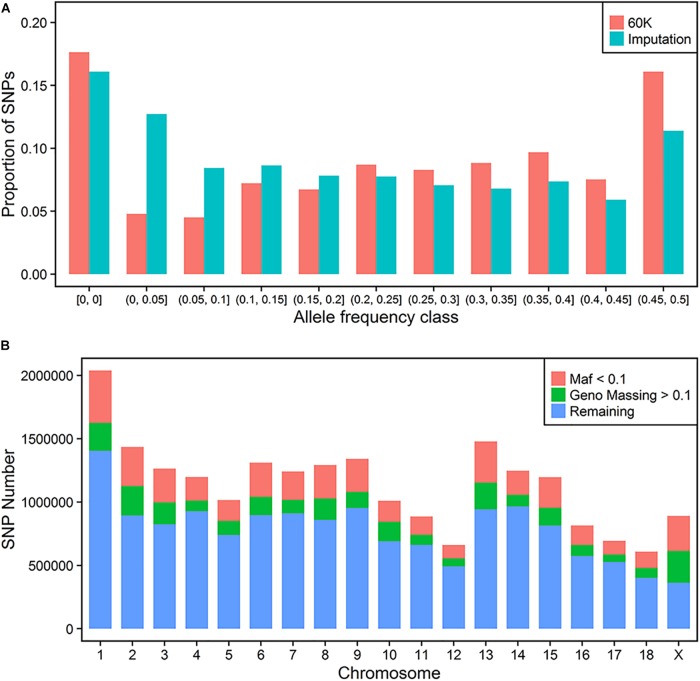
Imputation of whole-genome sequence variants. Distribution of SNP frequencies of 60 K and imputed variants across MAF classes **(A)**. Quality control for imputed variants with MAF and genotype missing rate **(B)**.

The distributions of proportion of the variants across MAF for 60 K and imputation results before QC are compared in Figure 1A. Both of them had a more than 15% homozygote rate in this population. In general, the imputed variants had a larger proportion of low frequency loci (below 0.2) when compared to 60K SNP classes. Finally, 1020 individuals with 40,057 sites were regarded as the target panel. More than 20 million variants were imputed for 1020 animals. After trimming the genotype probability with a threshold of 0.5, several variants contained missing values in *PED* files. We repeated the QC process using thresholds of missing rate of each variant and MAF. 2,572,745 variants with a low call rate (0.9) and 4,200,615 variants with a low MAF (0.01) were discarded further in the QC process. Thus, a total of 14,851,440 variants were retained for the following GWAS process (Figure 1B).

**FIGURE 2:**
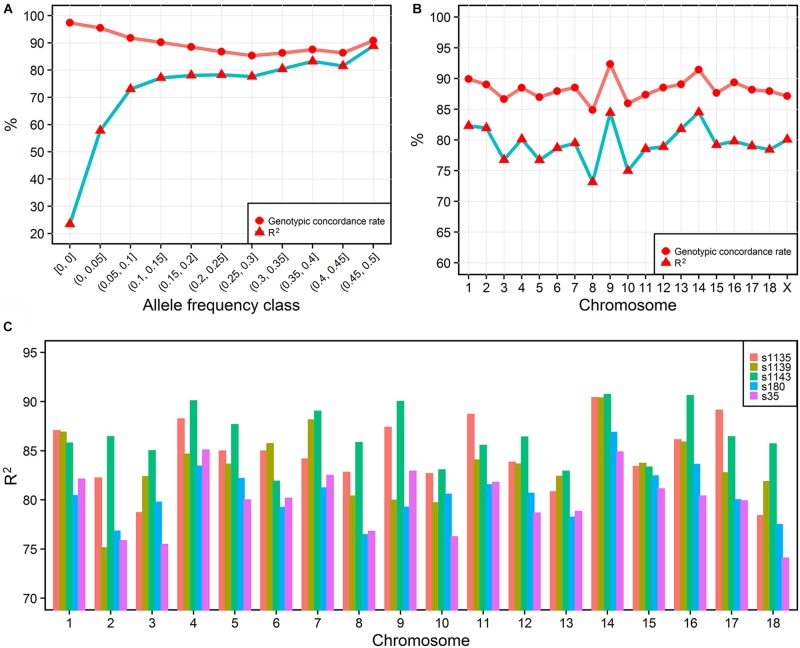
Imputation accuracy. The imputation accuracy from IMPUTE2 internal cross-validation across MAFs **(A)** and chromosomes **(B)**. *R^2^* is the squared correlation. The squared correlation between five sequenced and imputed individuals **(C)**.

To investigate the imputation accuracy across MAF in detail, we studied the genotypic concordance rate and *R^2^* according to MAF and chromosome (Figures 2A,B). Genotypic concordance rate and *R^2^* between the best-guess imputed and true variants in the cross-validation reached an average of 89 and 80%, respectively. With the increasing of MAF, *R^2^* increased from 23 to 88%, while genotypic concordance rate decreased from 97% to approximately 86% (Figure 2A). The whole-genome genotypic concordance rate fluctuated between 85 and 92%, with *R^2^* fluctuating between 73 and 85%, and varying across chromosomes (Figure 2B). *R^2^* between variants in five sequenced individuals and best-guess imputed variants, fluctuated between 79 (s35) and 87% (s1143) across individuals (Figure 2C).

### Single-Trait GWAS

Totally, we identified 197 QTLs for all hematological traits at three different stages. Among them, we obtained 75, 86, and 36 QTLs for hematological traits at day 18, day 46, and at day 240, respectively. With regard to the three classes of hematological traits, we identified 108, 76, and 13 QTLs for erythroid parameters, leukocyte parameters, and platelet parameters, respectively. To detect the number of independent QTLs identified in this study, we defined a minimum distance of 5 Mb between two top SNPs to differentiate two independent QTLs. Thus, we identified 95 unique QTLs, 87 of which are novel compared to the 60 K GWAS in the same population. Furthermore, we identified a series of significant variants located on some annotated protein-coding genes.

### Loci for Erythrocyte Traits

**FIGURE 3:**
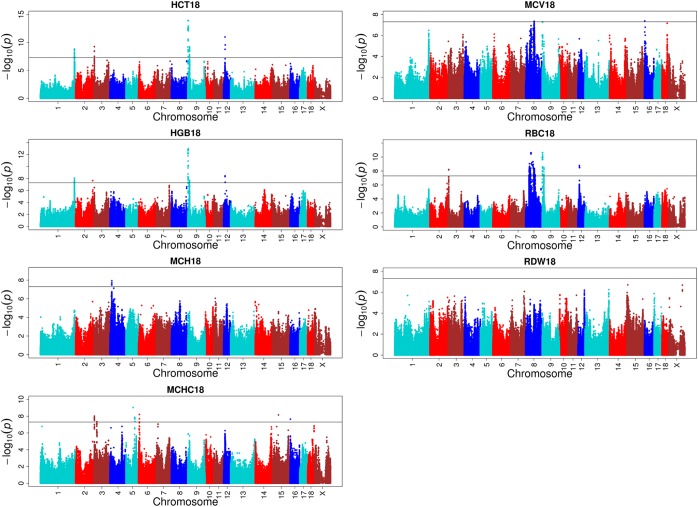
Single-marker GWAS results for seven erythrocyte traits at day 18. The *y*-axis and the *x*-axis represent the negative log_10_ *P-value* of the SNPs and the genomic positions separated by chromosomes, respectively, and the black solid lines indicate the significance threshold (negative log_10_ 5E-08).

One hundred and eight QTLs significantly associated with the seven erythrocyte traits in different stages were identified. The Manhattan plots for these association results and detailed information across the three different stages are presented in Figure 3, Table 1, Supplementary Figures S3, S4, and Supplementary Table S2. Among the 108 QTLs, 22, 68, and 18 QTLs were identified for days 18, 46, and 240, respectively. There is no significant variant association with RDW at day 18, and HGB and RBC at day 46 (*P* > 5E-08). Variants affecting HCT, HGB, and RBC at day 18 are located on SSC9 and near within 1 Mb, and are treated as identical QTLs (*P* = 1.18E-14, *P* = 1.01E-13, and *P* = 2.27E-11). The nearest gene in this region is the serine/threonine kinase 33 (*STK33*) gene. We also identified a QTL at 304,622,788 bp located on SSC1 that possibly affected HCT and HGB at day 18. The most significant SNP (*P* = 1.51E-09) was located on the far upstream element-binding protein 3 (*FUBP3*) gene. Several significant loci were detected for erythrocyte traits at day 46, especially for HCT, MCHC, and MCV (Supplementary Figure S3). Regarding the traits at day 240, one and two novel QTLs were detected for MCH and MCHC, respectively. And some QTLs located on SSCX were identified for MCV and RDW. The novel locus associated with MCH was located at ∼77 Mb on SSC11 including 23 significant SNPs (*P* < 5E-08). The most significant SNPs (*P* = 1.97E-09) were exactly located within the fibroblast growth factor 14 (*FGF14*) gene. The two novel QTLs associated with MCHC were located on SSC6 and SSC17, respectively. More information about significant SNPs are listed in Table 1 and Supplementary Table S2.

**Table 1:** Genome-wide significant loci associated with erythroid traits at day 18 obtained by single-trait GWAS.

Trait^1^	Chr^2^	Pos (bp)^3^	*P*-value	Num^4^	Nearest gene^5^	Dis (bp)^6^	Maf^7^
HCT18	1	304,622,788	1.51E-09	297	*FUBP3*	Within	0.04
HCT18	3	5,110,712	5.65E-10	86	*USP42*	4297	0.019
HCT18	9	1,131,862	1.18E-14	179	*STK33*	8983	0.018
HCT18	12	12,753,349	1.07E-11	7	*AXIN2*	91,972	0.015
HGB18	1	304,622,788	8.41E-09	210	*FUBP3*	Within	0.041
HGB18	2	152,599,639	2.16E-08	2	*NA*	NA	0.012
HGB18	9	3,329,737	1.01E-13	157	*OR6A2*	183684	0.022
HGB18	12	12,995,699	3.54E-09	7	*APOH*	146651	0.017
MCH18	4	16,730,041	1.17E-08	14	*WDYHV1*	Within	0.146
MCHC18	3	5,155,853	9.79E-09	156	*EIF2AK1*	Within	0.019
MCHC18	3	27,061,999	4.64E-08	5	*TMC7*	57,656	0.011
MCHC18	5	63,702,557	9.37E-10	490	*BCL2L14*	Within	0.014
MCHC18	6	7,942,824	6.17E-09	6	*CDYL2*	247,063	0.047
MCHC18	15	53,936,604	7.10E-09	1	*KLKB1*	Within	0.017
MCHC18	16	3,021,631	2.26E-08	1	*NA*	NA	0.013
MCV18	8	76,425,157	4.47E-08	25	*SHROOM3*	22105	0.502
MCV18	16	3,226,563	4.13E-08	1	*NA*	NA	0.02
RBC18	3	5,110,712	6.09E-09	3	*USP42*	4297	0.019
RBC18	8	47,538,823	2.50E-11	1222	*PDGFC*	136,724	0.251
RBC18	8	66,311,823	4.69E-10	2183	*TECRL*	Within	0.28
RBC18	9	1,131,862	2.27E-11	176	*STK33*	8983	0.018
RBC18	12	12,753,349	1.65E-09	7	*AXIN2*	91,972	0.015

*^1^Abbreviations of hematological traits, i.e., HCT18 is hematocrit at day 18. ^2^Chromosomal locations of the most significant SNPs. ^3^Positions of the most significant SNPs according to sus scrofa 10.2 genome assembly. ^4^The number of SNPs that reached the threshold (P < 5E-08). ^5^The nearest annotated genes from the most significant SNPs. ^6^The distance from the most significant SNPs to the nearest genes. ^7^The frequency of the most significant SNPs*.

### Loci for Leukocyte Traits

Seventy six loci significantly associated with the seven leukocyte traits at different stages were detected. The related Manhattan plots and detailed information across three different stages are presented in Supplementary Figures S5–S7 and Supplementary Table S2. Among the 76 loci, 47, 11, and 18 QTLs were identified to be responsible for the seven leukocyte traits at the three different stages, respectively. At day 18, many QTLs were identified for MON, MONR, and WBC. One locus near the *FGF14* gene located on SSC11 was simultaneously associated with GRAN, LYM, and WBC at day 18. There was only one possible QTL located on SSC8 that affected GRAR at day 18. This region held 35 significant SNPs, where the most significant SNP was at the position of 10,485,550 bp and located in the coiled-coil and c2 domain containing 2A (*CC2D2A*) gene. At day 46, the majority of QTLs were identified associated with MONR. At day 240, some identical QTLs were uncovered for MON and MONR. There is no significant association for lymphocyte count (LYM) and WBC (*P* > 5E-08). The detailed descriptions of significant SNPs across the three different stages of QTLs are presented in Supplementary Table S2.

### Loci for Platelet Traits

Unlike the plenty of loci identified for erythrocyte and leukocyte parameters, only a few loci were identified for platelet traits (Supplementary Figure S8 and Supplementary Table S2). Six and seven loci were associated with platelet traits at days 18 and 46, respectively. There was no significant association with platelet traits at day 240. Although some SNPs were significantly associated with MPV at day 18, no annotated gene was found around a distance of 700 Kb near the most significant SNP (*P* = 4.44E-10). For PDW at day 18, a region of 1.56 Mb on SSC5 was identified including 13 significant SNPs, and the most significant SNP (*P* = 2.2E-08) was located in the activating transcription factor 7 interacting protein (*ATF7IP*) gene. Fourteen significant SNPs located on SSC11 were detected for PDW at day 46. The most significant SNP (*P* = 1.19E-09) was located in the integrin subunit beta like 1 (*ITGBL1*) gene.

### Multitrait GWAS

**FIGURE 4:**
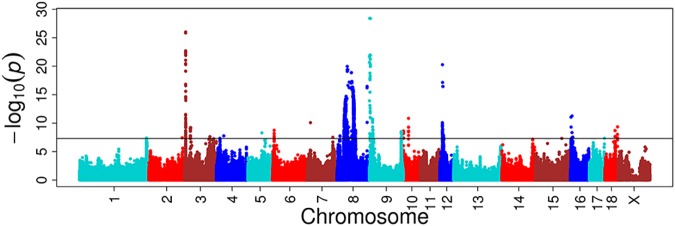
Multitrait GWAS results for correlated hematological traits at day 18. The multiple traits include HCT, HGB, MCH, and MCV at day 18. The *y*-axis and the *x*-axis represent the negative log_10_ *P-value* of the SNPs and the genomic positions separated by chromosomes, respectively, and the black solid lines indicate the significance threshold (negative log_10_ 5E-08).

For these four multitrait GWAS of correlated traits, we identified eight, one, four, and three novel significant loci, respectively, compared to single-trait GWAS (Figure 4, Supplementary Figure S9 and Supplementary Table S3). The multitrait GWAS (including HCT, HGB, MCH, and MCV traits) at day 18 identified two, one, two, one, and two loci located on SSC3, SSC5, SSC7, SSC9, and SSC10, respectively (Supplementary Table S3). The most significant SNP (*P* = 2.33E-08) was located on SSC3 at 115 Mb near to an annotated gene called the lysocardiolipin acyltransferase 1 (*LCLAT1*) gene. The other significant SNPs are listed in Supplementary Table S3.

## Discussion

### Genotype Imputation

In the current study, we obtained 14,851,440 variants with a high imputation accuracy. Genotypic concordance rate and R^2^ between the best-guess imputed and true variants in the cross-validation reached an average of 89 and 80%. In the case of the pig, most studies focused on the imputation from low-density genotypes to 60 K variants with correlations ranging from 0.938 to 0.992 for imputation from 3 to 60 K (Cleveland and Hickey, 2013), and with R^2^ ranging from 0.88 to 0.95 for imputation from 9 to 60 K (Badke et al., 2014). However, few studies reported the imputation accuracy from 60 K to a whole-genome sequence in the pig. Genotypic concordance rate was reported to be 92.1% from 80 to 650 K and 85.6% from 650K to whole-genome sequence variants using a stepwise imputation strategy in 1,363 Duroc pigs (Zhang et al., 2018), and was lower compared to the present study (89%). Another study showed an average correlation of 80% between the true and imputed genotypes ranging from 0.74 to 0.86 with imputing 60K to whole-genome sequence variants in Sutai pigs (Yan et al., 2017). Sometimes, comparing the imputation accuracy among different studies is difficult since it is defined in different ways. Genotypic concordance rate is highly sensitive to MAF and is not appropriate for comparing genotypes with different MAF. To directly compare other studies, we adopted both genotypic concordance rate and R^2^ to estimate imputation accuracy.

Many factors affect the accuracy of imputation from low- or medium-density genotypes to whole-genome sequence data. As the whole-genome sequence data contains a huge percent of low MAF variants compared to chip data, imputation accuracy will be affected by those low MAF variants more in the imputation to whole-genome sequence data. In the present study, R^2^ decreased from 72 to 22% when the MAF decreased from 0.1 to 0. In other studies, the same trend was found (Daetwyler et al., 2014; van Binsbergen et al., 2014). The relationships between the target and reference panels is another key factor that influences imputation accuracy (van Binsbergen et al., 2014). We sequenced 19 ancestors of F_2_ to achieve a high imputation accuracy. LD and reference size will also affect the imputation accuracy according to many studies (van Binsbergen et al., 2014). Usually, a smaller distance between markers in the target panel and in reference would lead to a higher imputation accuracy. Thus, imputation accuracy can be improved by considering different factors, which will lead to a high accuracy in GWAS and genomic selection.

### Whole-Genome Association vs. 60K GWAS and Potential Novel Candidate Genes

With the development of advanced statistical models and the decrease of genotyping cost, GWAS has become a considerably effective and popular method to search for the association between the genetic variants and complex traits across the whole genome (Hirschhorn and Daly, 2005). Previously, by performing a single-marker GWAS and a LONG-GWAS in the same population of 18 hematological traits at three different stages using PorcineSNP60 BeadChip (Zhang et al., 2013), we identified 38 genome-wide significant regions distributed on SSC1, 4, 5, 7, 8, 10, 11, 12, 13, 17, and 18, which only confirmed 10 QTLs (Supplementary Figure S10 and Supplementary Table S4) from the linkage mapping result obtained using 194 microsatellites for traits of seven erythroid parameters at three stages, and five leukocyte and four platelet parameters at day 240 within the same population (Zou et al., 2008; Yang et al., 2009). Because of the relatively shorter LD (10.5 kb) decay distance (threshold: *r*^2^ = 0.3) in most native pigs than in western pigs (Ai et al., 2013), and the density of the current 60 K BeadChip cannot effectively cover the LD block (∼80 kb), if there is no marker in tight LD with casual variants in a such broader region, no significant association signal would be detected as LD decreased rapidly. Thus, some QTLs were missing when we conducted the 60 K GWAS. In comparison, the whole-genome sequence including almost genetics variants across genome as well as causative mutation for traits of interesting association between causative variants and phenotypes can meet the shortcoming of low-density BeadChip based association.

After performing the whole-genome association study, we identified 108, 76, and 13 QTLs for erythroid, leukocyte, and platelet traits, respectively. Among these QTLs, 95 QTLs are unique and 87 QTLs are novel when compared to the 60K GWAS result in the same population (Supplementary Table S4), of which 39, 41, and 7 are novel QTLs for erythroid, leukocyte, and platelet traits, respectively. These novel QTLs distributed on almost all chromosomes except chromosome 18 across the whole genome. Because of the numerous novel results, we herein paid more attention to the gene which is function-related to each trait or the gene which the most significant SNP is rightly located on for each trait. We identified one locus that affected LYM and WBC at day 18 with the most significant position of 50, 736, and 762 bp (*P_LY M_* = 2.03E-10 and *P_WBC_* = 7.10E-10), which is near the *TOX2* gene. *TOX2* is a transcription factor that shares a highly conserved high-mobility group DNA-binding domain with the other TOX family members. A recent study shows that *TOX2* can regulate the development of natural killer cells with the control in T-BET expression (Vong et al., 2014). Another study illustrates that TOX is a requirement for the differentiation of common lymphoid progenitors into innate lymphoid cells *in vivo* (Seehus et al., 2015). These evidences imply that *TOX2* gene may be a candidate gene for WBC and LYM at day 18. Furthermore, except the QTL on SSC8, we identified another novel QTL located on SSC11 associated with MCH at day 240. This region including 23 significant SNPs, with the top SNP located at the position of 77,754,820 bp (MAF = 0.212, *P* = 1.97E-09) and posited the fibroblast growth factor 14 (*FGF14*) gene. Fibroblast growth factors (FGFs) family are involved in a variety of biological processes. Among the *Fgf* family, *FGF14* is one member of the intracellular subfamily, also known as iFGFs (Itoh and Ornitz, 2008). The iFGFs function as intracellular proteins in an FGFR-independent way (Wang et al., 2000; Goldfarb, 2005). *FGF14* was identified to be associated with neuronal signaling in mice (Wang et al., 2002). Recent studies on the *FGF14* gene focused on neurology in humans and in mice (Amado et al., 2017; Di Re et al., 2017; Hsu et al., 2017). Interestingly, this gene was also identified to be a candidate for teat number in Duroc pigs (Tan et al., 2017), implying the pleiotropy of *FGF14*. These new candidate genes could help to complete the gene regulatory network of hematological traits.

We almost confirmed all the QTLs identified in our prior 60K GWAS based on a single-marker, except the QTLs located on SSC5, SSC7, and SSC13 for MCH at day 18, WBC at day 240, and PDW at day 46. The undetected signal is probably because of the higher threshold in the whole-genome GWAS. In particular, as to the QTLs located on SSC7 or SSC8 for HCT, MCH, and MCV at day 240, respectively, we did not discuss these QTLs in detail here, because the conditional GWAS was performed and results have been elaborately discussed by Zhang et al. (2013) in the same population using SNP60 BeadChip data. Although we identified many novel QTLs, most of them are time-specific except for the region on SSC8 harboring the kit proto-oncogene receptor tyrosine kinase (*KIT*) gene, implying that the same hematological trait at different stages may be affected by different genes.

### Result Consistency With Other Studies

To the best of our knowledge, there are only six published studies demonstrating the association between either all 18 hematological traits or part of these traits and genomic loci using the Porcine SNP60 BeadChip based on GWAS in pigs (Luo et al., 2012; Wang et al., 2013; Zhang et al., 2013, 2014; Jung et al., 2014; Ponsuksili et al., 2016). The *KIT* gene was proposed as a candidate gene for hematological traits by some different studies. We found that the candidate gene of *KIT* should be responsible for the MCV trait (days 18, 46, and 240), and MCH and RBC traits (day 240) in our previous study (Zhang et al., 2013). The same result was obtained in Landrace × Korean native pigs F_2_ population at the age of 140 days (Jung et al., 2014). However, Luo et al. only identified the candidate gene of *KIT* associated with two erythroid traits including MCH and MCV at day 240 in White × Minzhu F_2_ population (Luo et al., 2012). In this study, we found that this candidate gene can also significantly affect the PDW trait at day 18, although the SNPs in that region did not reach the significant threshold (*P* = 1.35E-07). The relatively low significance may be because of the small samples for this trait, i.e., only 250 samples that held phenotypes were used for GWAS. If we can increase number of the valid samples, the association between PDW and the genotypes may emerge. Beside the *KIT* gene claimed as the candidate gene for hematological traits in majority studies at different stages (i.e., days 140 and 240), some other candidate genes were identified, including neuroligin 4, x-linked (*NLGN4X*) and high-mobility group box family member 2 (*TOX2*) gene, which was identified in the Commercial Landrace population (Ponsuksili et al., 2016). In the GWAS study in the Commercial Landrace population, Ponsuksili et al. (2016) identified a candidate gene named *NLGN4X* associated with MCH, MCV, and RDW traits at an average day 170 using the Bayesian GWAS approach. The mutations of *NLGN4X* are generally linked with autism and Asperger syndrome in different human populations (Jamain et al., 2003; Volaki et al., 2013; Landini et al., 2016). We found that this *NLGN4X* gene is not only associated with MCV traits at day 46 but also associated with HCT, MCHC, and PCT at day 46. In summary, although we have confirmed the associations of different QTLs or candidate genes with these hematological traits reported in other studies, the different genetic backgrounds and populations and the different stages when the traits are measured would lead to different association results.

### Multitrait GWAS

A multivariate linear mixed model has been recently considered as a remarkably efficient method in GWAS, not only because of its stronger ability in correcting sample relatedness (Meyer, 1991; Amos, 1994; Korte et al., 2012) and population stratification (Yu et al., 2006), but also because of the advantage over standard univariate analysis in terms of increasing statistical power (Korte et al., 2012). Multivariate analysis can increase detection power by accumulating common genetic factors together for multiple correlated traits and increasing the sample size (Stephens, 2013). We thus used multiple correlated traits implemented in a multitrait linear mixed model to discover more novel QTLs. The genetic correlation and the phenotype correlation are highly similar. The statistical power of multitrait GWAS on HCT, HGB, MCH, and MCV at day 18 were increased in most chromosomes. The novel loci with the most significant SNP at the position of 115,277,228 bp (*P* = 2.33E-08) located on SSC3 near the lysocardiolipin acyltransferase 1 (*LCLAT1*) gene were found to be responsible for MCH and MCV at day 18. A study reported that *LCLAT* played an important role in the development of hematopoietic and endothelial lineages, probably acting at the level of the hemangioblast in mice (Wang et al., 2007).

## Conclusion

Whole-genome association analyses based on imputed sequence variants increase the possibility to identify more QTLs and the potential causative mutation. Multitrait GWAS has been proven to be a remarkably powerful statistical method to uncover the variants that affect multiple correlated traits. This study identified more than 87 QTL novel regions that affected 18 hematological traits at three different stages by single and multitrait GWAS, based on the whole-genome imputation data. We propose that the *FGF14* and *LCLAT1* genes located at SSC11 and SSC3 may affect MCH at day 240 and MCH as well as MCV at stage at day 18, respectively.

## Data Availability

The genotypic data of 933 F_2_ individuals analyzed for this study can be found in the Dryad Digital Repository (https://doi.org/10.5061/dryad.7kn7r) (Ma et al., 2013). The raw reads of the whole-genome sequence can be found from the NCBI sequence read archive (SRA) under the accession codes SRA065461 (Ai et al., 2015), SRP047260 (Moon et al., 2015), and SRP159212.

## Author Contributions

LH and ZZ conceived and designed the experiments. TG, GY, ZZ, and SX performed the experiments. GY, ZZ, FZ, TH, and YL analyzed the data. SX, WXi, and WXu contributed to materials and analysis tools. GY, TG, ZZ, and LH wrote the manuscript. All authors read and approved the final manuscript.

## Conflict of Interest Statement

The authors declare that the research was conducted in the absence of any commercial or financial relationships that could be construed as a potential conflict of interest.

## Footnotes

1 http://broadinstitute.github.io/picard/

2 http://www.well.ox.ac.uk/cfreeman/software/gwas/gtool.html
