# Supplementary material for: Imputation-Based Whole-Genome Sequence Association Study Reveals Constant and Novel Loci for Hematological Traits in a Large-Scale Swine F2 Resource Population
Source: Front Genet. 2018 Oct 22;9:401. doi: 10.3389/fgene.2018.00401 (PMC6204663; doi:10.3389/fgene.2018.00401)
Supplement: Supplementary file 1 [file Data_Sheet_1.DOCX]

Supplementary Material

# Imputation-Based Whole-Genome Sequence Association Study Reveals Constant and Novel Loci for Hematological Traits in a Large-scale Swine F_2_ Resource Population

Guorong Yan†, Tianfu Guo†, Shijun Xiao, Feng Zhang, Wenshui Xin, Tao Huang, Wenwu Xu, Yiping Li, Zhiyan Zhang*, Lusheng Huang*

State Key Laboratory for Pig Genetic Improvement and Production Technology, Jiangxi Agricultural University, 330045, Nanchang, P.R. China

* Corresponding author [bioducklily@hotmail.com](mailto:bioducklily@hotmail.com) (Z. Y. Zhang); [lushenghuang@hotmail.com](mailto:lushenghuang@hotmail.com)(L. S. Huang)

† these authors contributed equally to this work

Email addresses of authors:

Guorong Yan: [guorongyan@outlook.com](mailto:guorongyan@outlook.com)

Tianfu Guo: [guotianfu2001@163.com](mailto:guotianfu2001@163.com)

Shijun Xiao: [shjx_jxau@hotmail.com](mailto:shjx_jxau@hotmail.com)

Feng Zhang: [297946506@qq.com](mailto:297946506@qq.com)

Wenshui Xin: [xinwenshui@yeah.net](mailto:xinwenshui@yeah.net)

Tao Huang: [taohuang@live.cn](mailto:taohuang@live.cn)

Wenwu Xu: [2274926981@qq.com](mailto:2274926981@qq.com)

Yiping Li: [571789240@qq.com](mailto:571789240@qq.com)

Zhiyan Zhang: [bioducklily@hotmail.com](mailto:bioducklily@hotmail.com)

Lusheng Huang: [lushenghuang@hotmail.com](mailto:lushenghuang@hotmail.com)

**Supplementary Figures and Tables**

**Supplementary Figures**

**
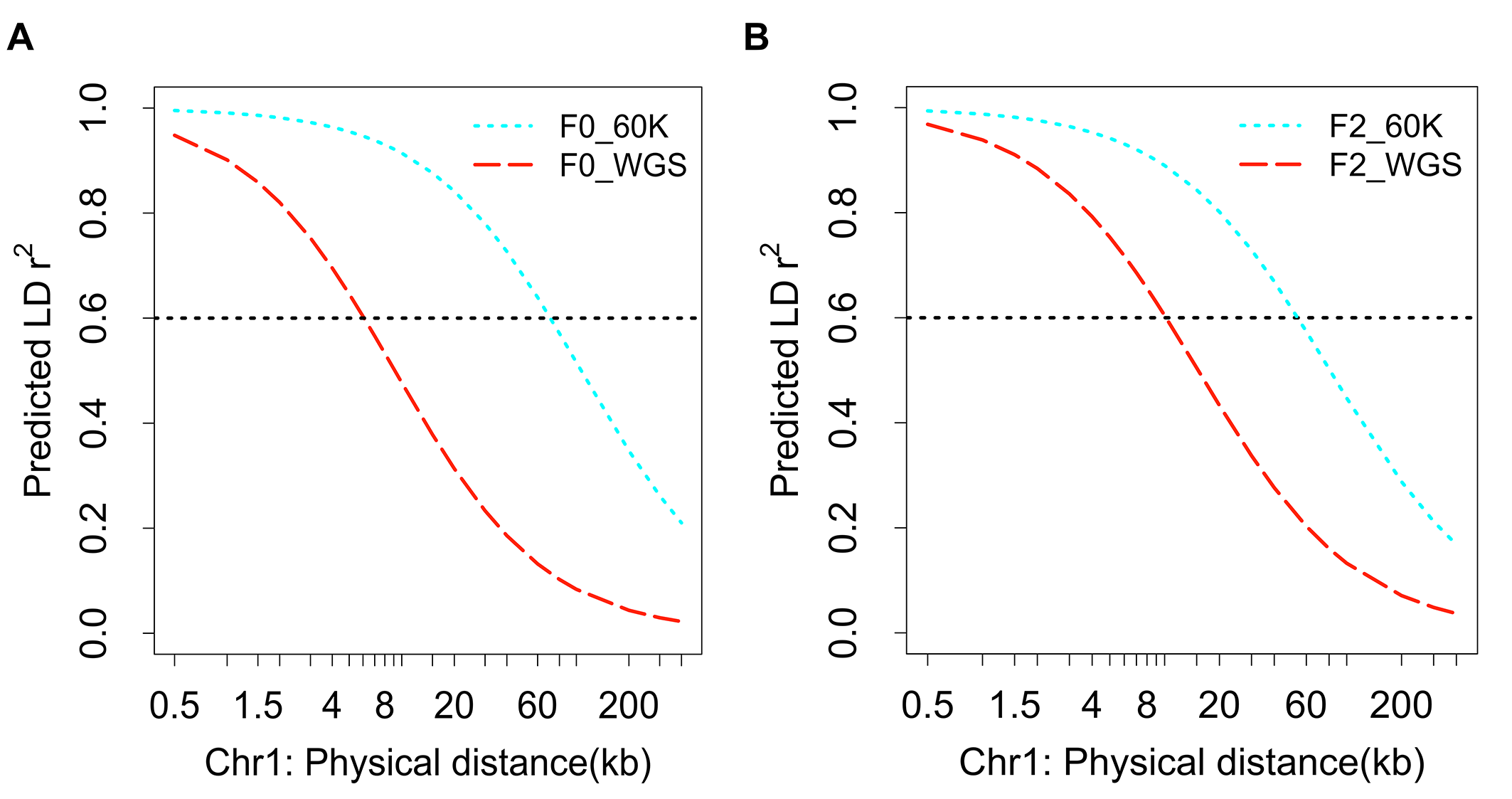
**

**Supplementary Figure S1. Linkage disequilibrium (LD) decay for F0 and F2 individuals in chromosome 1.** LD decay was calculated for F0 using 60K genotype (*light blue line*) and whole-genome imputed data (*red line*) (**A**). LD decay was calculated for F2 using 60K genotypes (*light blue line*) and whole-genome imputed data (*red line*) (**B**). X-axis indicates the physical distance (kb) between two markers, and if distances between two markers is greater than 400kb, the LD will not be calculated. Y-axis indicates the predicted LD by non-linear fit.

**
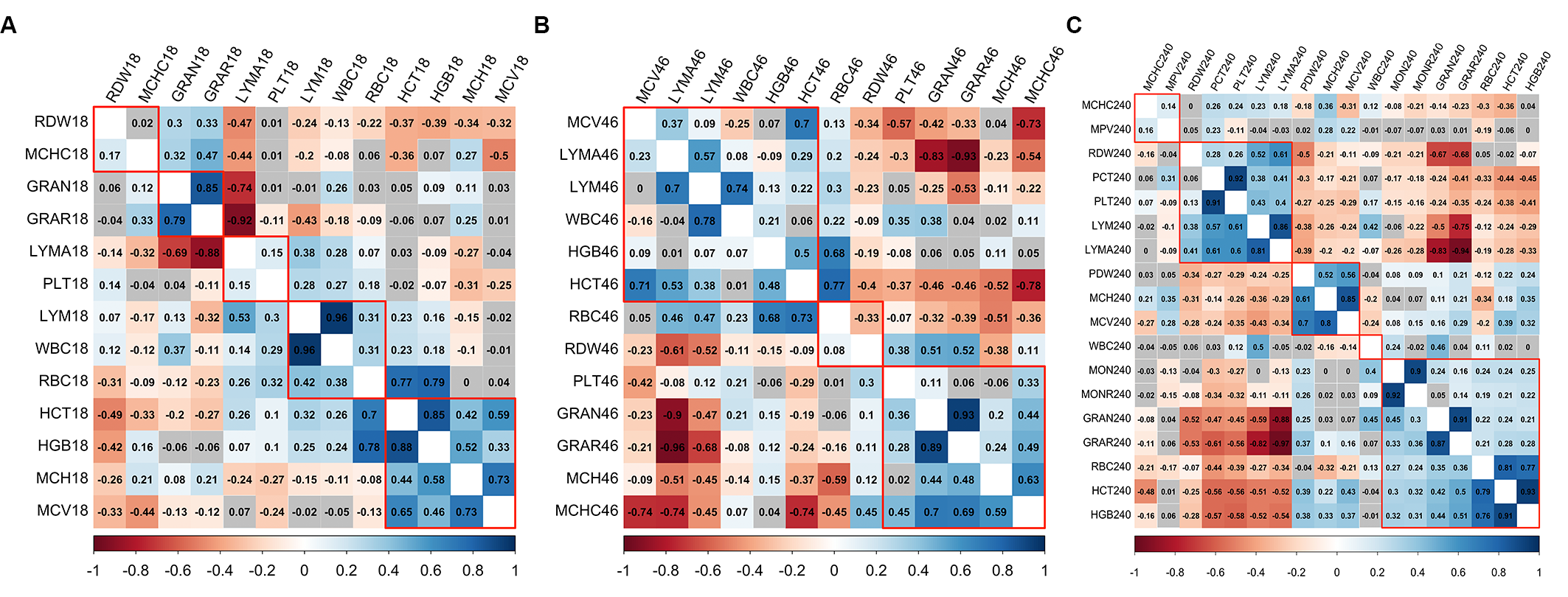
**

## Supplementary Figure S2. Phenotypic and genetic correlations for traits from different stages. Figure shows correlations in day-18(A), day-46(B) and day 240 (C). The values in the upper and lower triangles represent genetic correlation and phenotypic correlation, respectively. Progressive shades of *blue* and *red* refer to positive and negative correlation coefficients, respectively. Shades of *grey* indicate insignificant correlations. The numbers in every grid are correlation coefficients. Traits in red rectangles indicate that they are a cluster, which are clustered by hierarchical clustering algorithm.


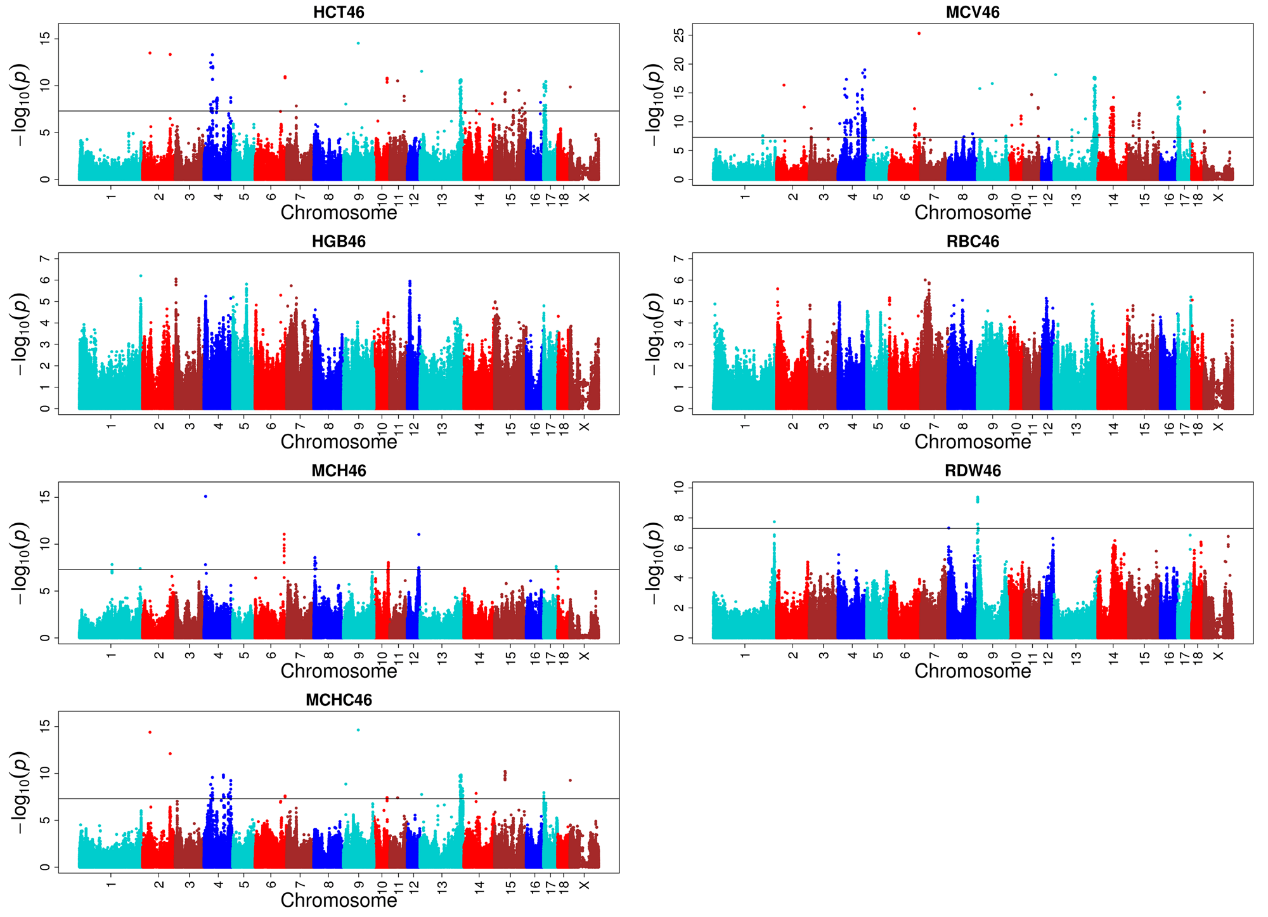


## Supplementary Figure S3. Single-marker GWAS results for seven erythrocyte traits at stage of day-18. The y-axis and x-axis represent the negative log_10_ *P value* of the SNPs and the genomic positions separated by chromosomes, respectively, and the black solid lines indicate the significance threshold (negative log_10_ 5E-08).


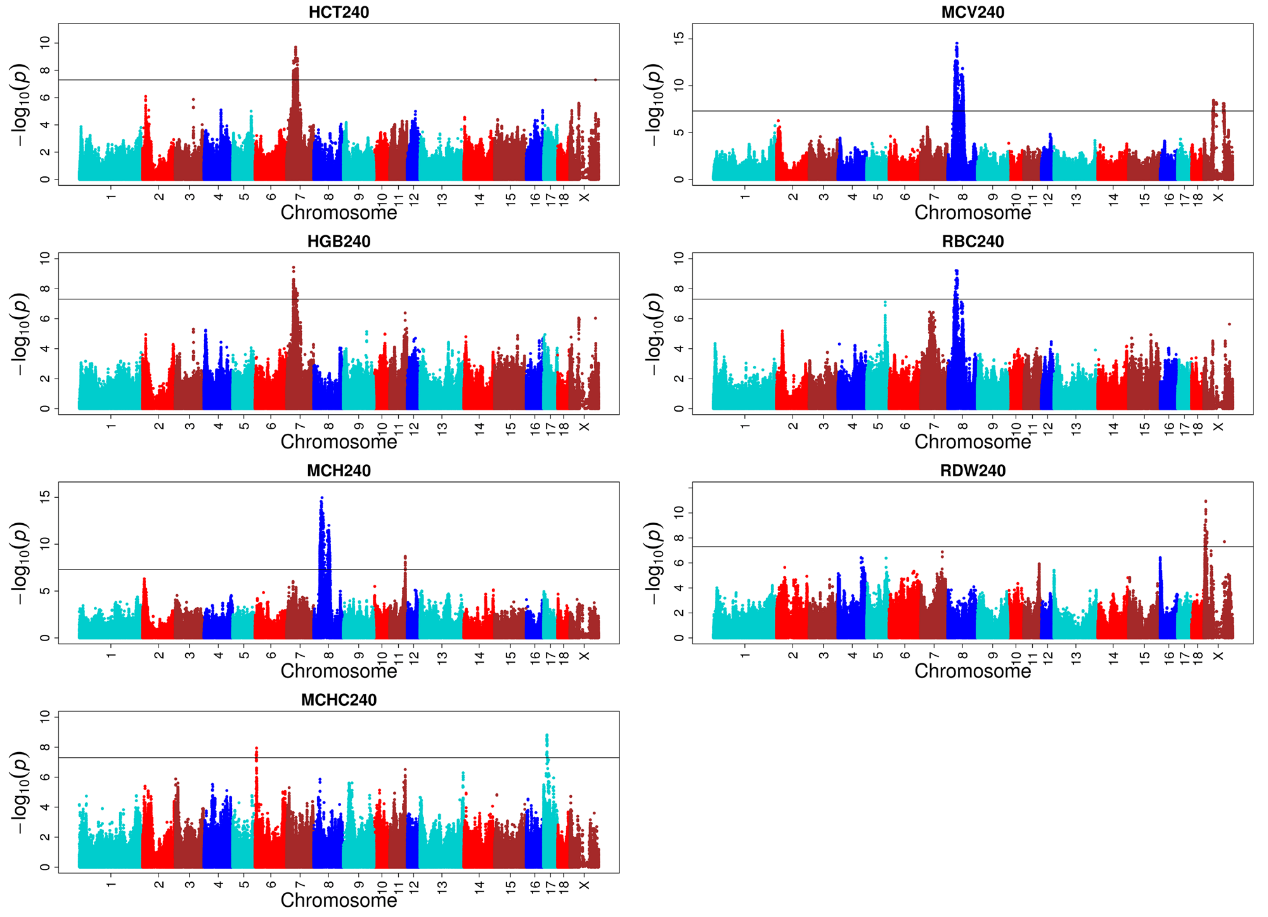


## Supplementary **Figure S4. Single-marker GWAS results for seven erythrocyte traits at stage of day-240.** The y-axis and x-axis represent the negative log_10_ *P value* of the SNPs and the genomic positions separated by chromosomes, respectively, and the black solid lines indicate the significance threshold (negative log_10_ 5E-08).


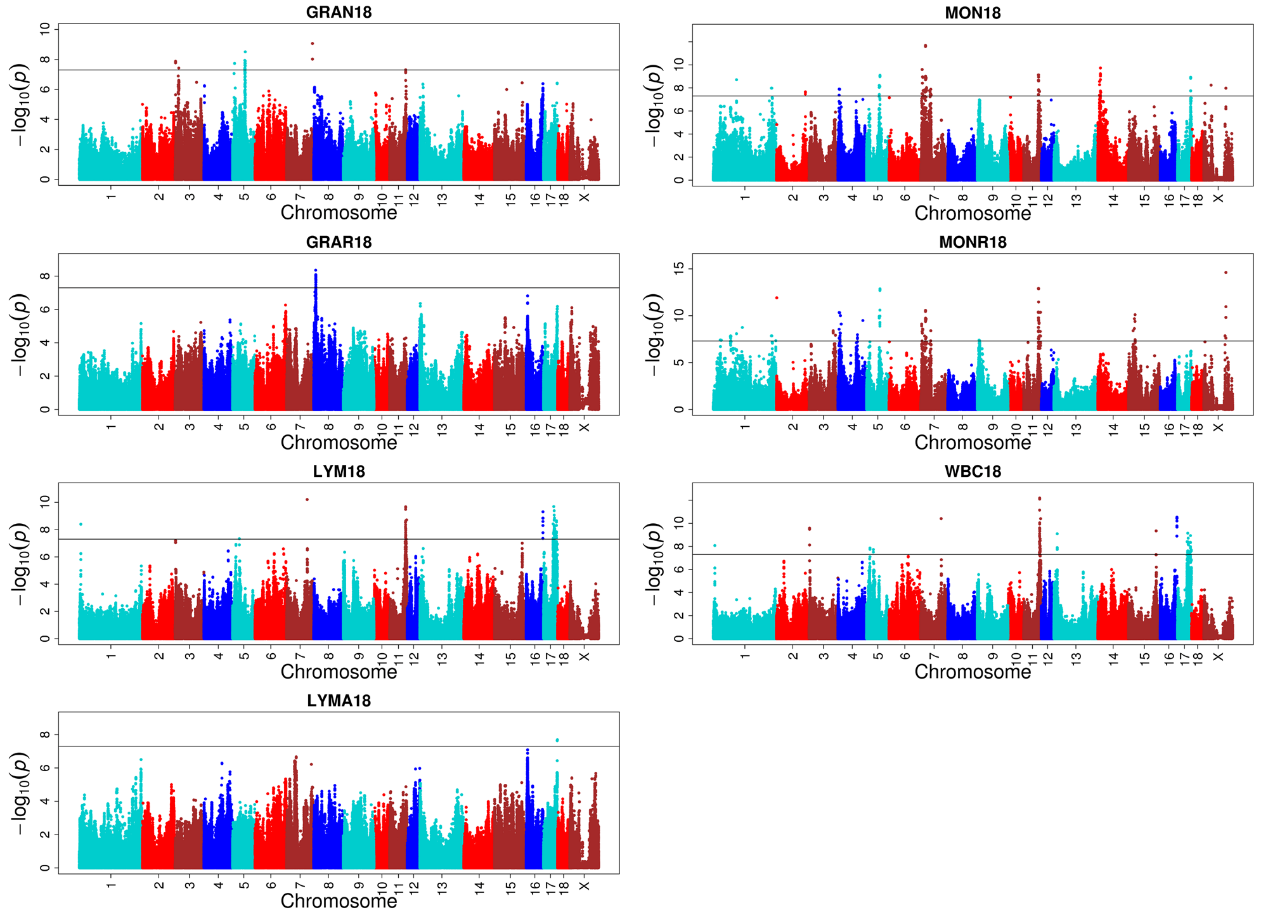


## Supplementary **Figure S5.** **Single-marker GWAS results for seven leukocyte traits at stage of day-18.** The y-axis and x-axis represent the negative log_10_ *P value* of the SNPs and the genomic positions separated by chromosomes, respectively, and the black solid lines indicate the significance threshold (negative log_10_ 5E-08).


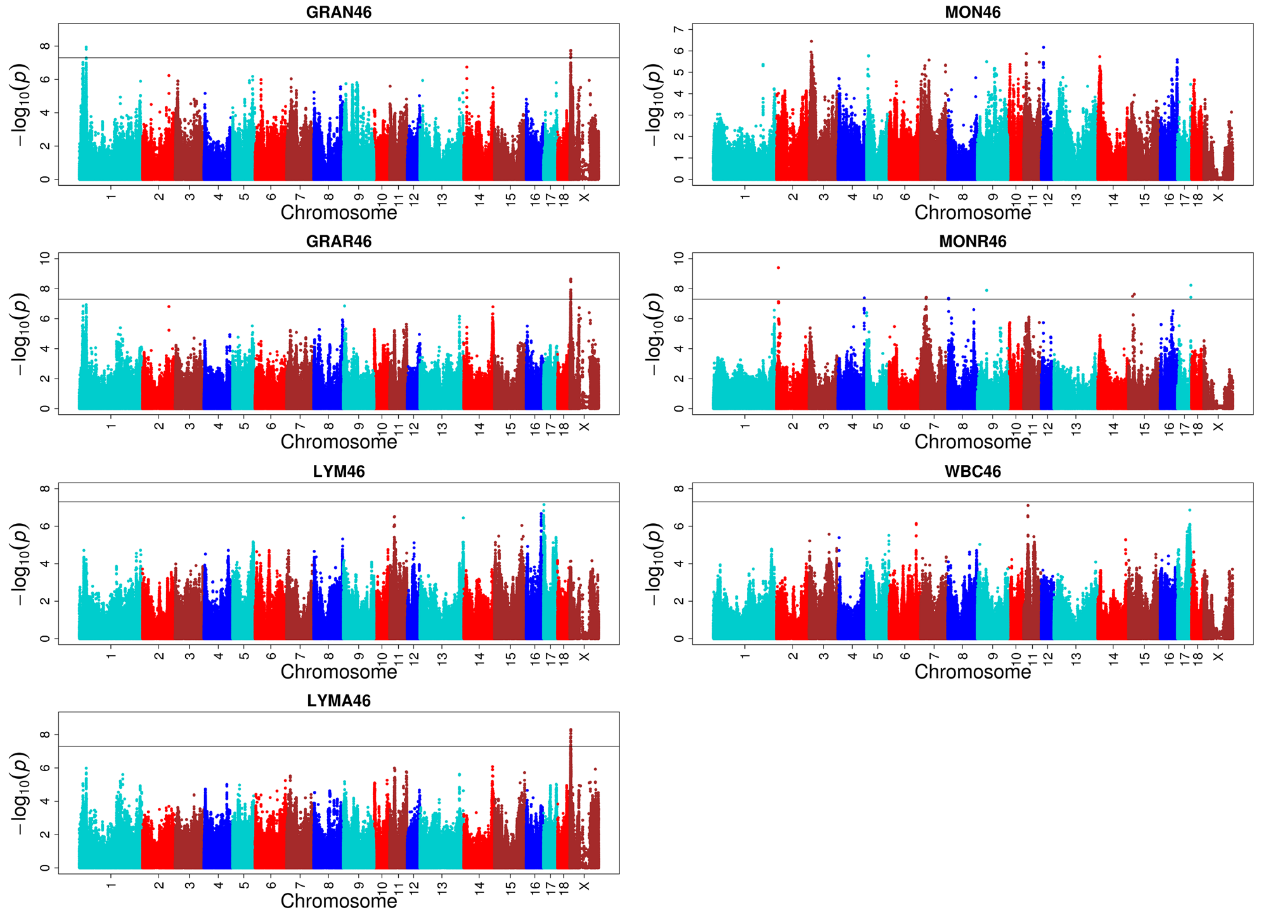


## Supplementary **Figure S6.** **Single-marker GWAS results for seven leukocyte traits at stage of day-46.** The y-axis and x-axis represent the negative log_10_ *P value* of the SNPs and the genomic positions separated by chromosomes, respectively, and the black solid lines indicate the significance threshold (negative log_10_ 5E-08).


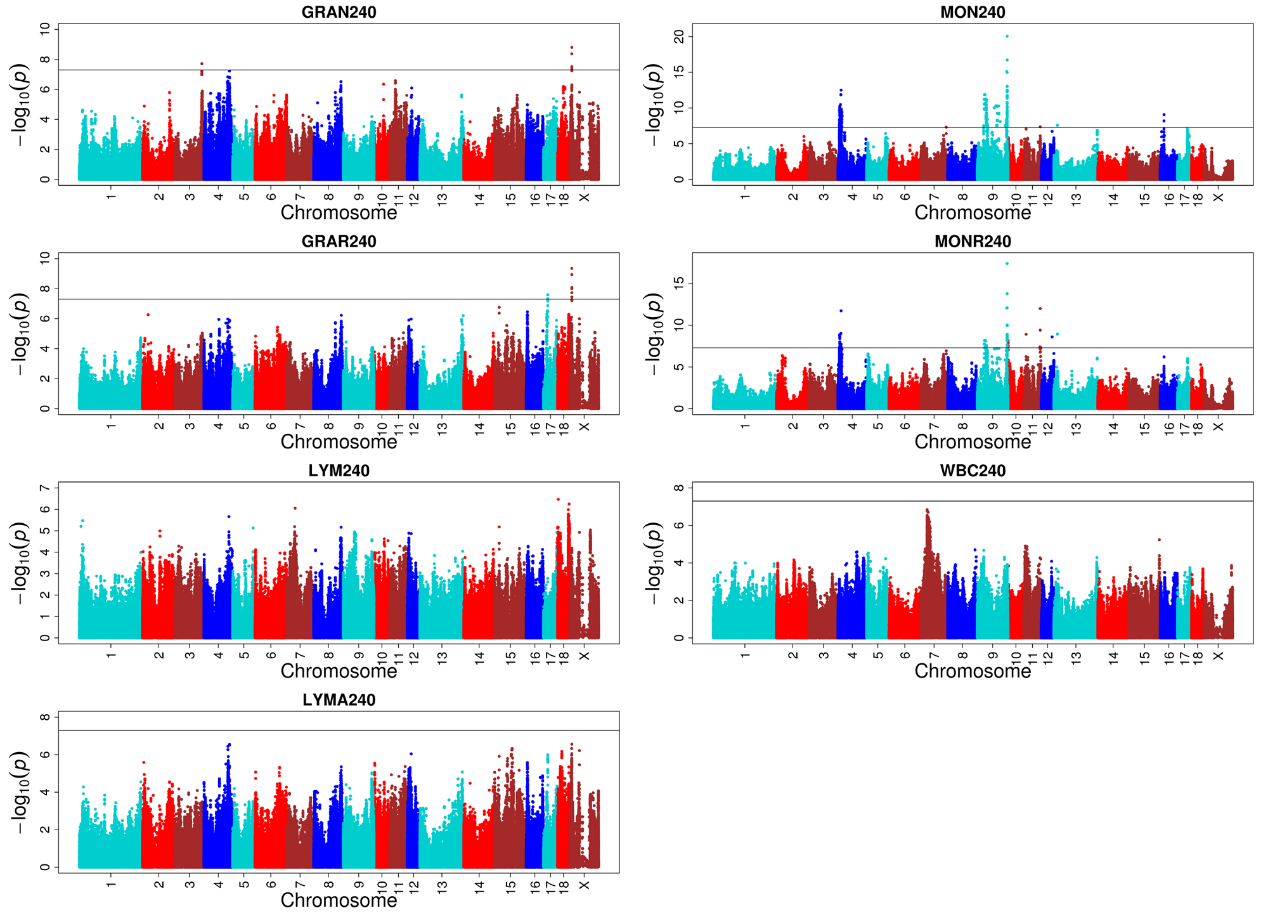


## Supplementary **Figure S7.** **Single-marker GWAS results for seven leukocyte traits at stage of day-240.** The y-axis and x-axis represent the negative log_10_ *P value* of the SNPs and the genomic positions separated by chromosomes, respectively, and the black solid lines indicate the significance threshold (negative log_10_ 5E-08).


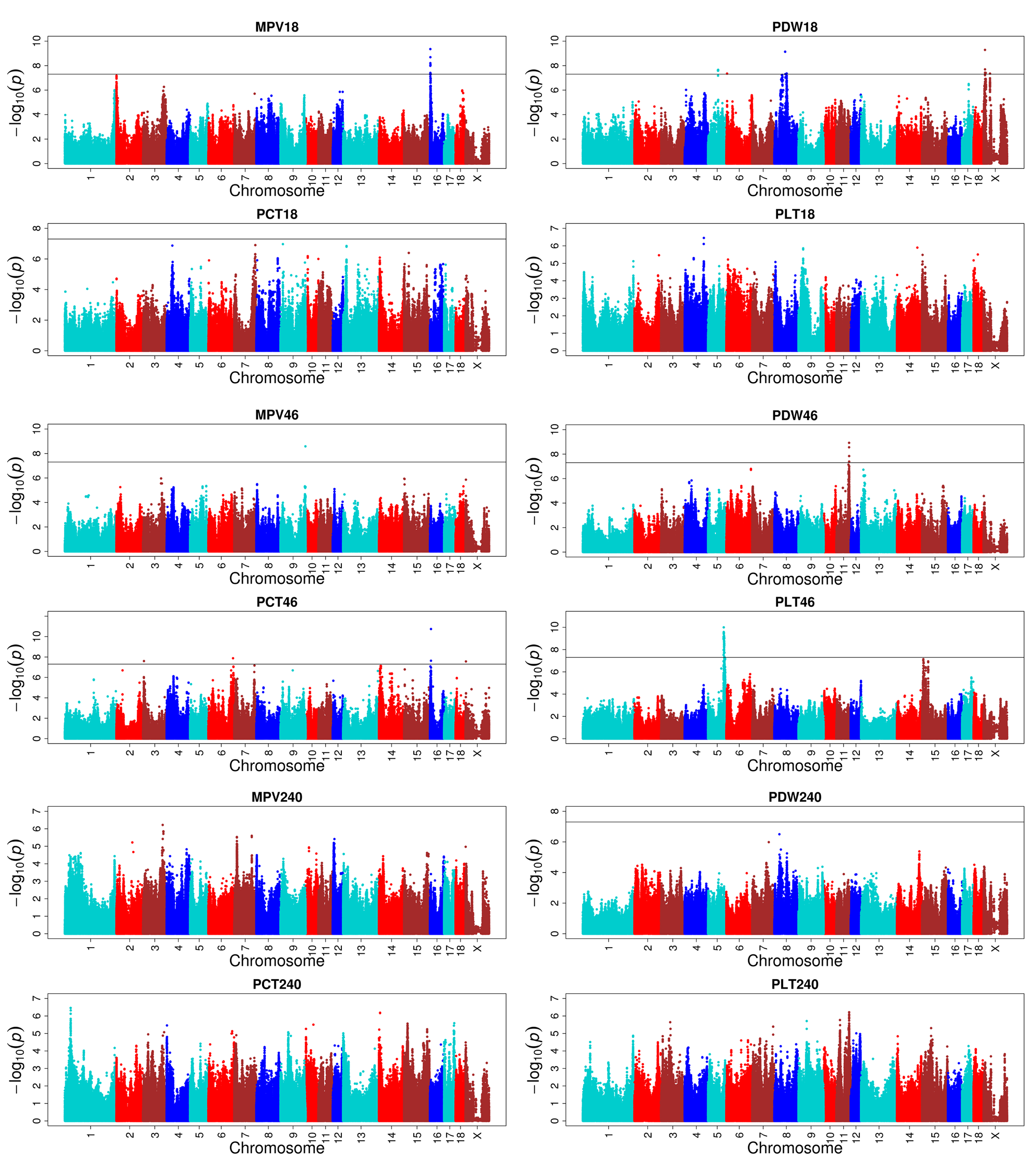


## **Supplementary Figure S8.** **Single-marker GWAS results for four platelet traits at age of day-18, day-46 and day-240.** The y-axis and x-axis represent the negative log10 P value of the SNPs and the genomic positions separated by chromosomes, respectively, and the black solid lines indicate the significance threshold (negative log10 5E-08).


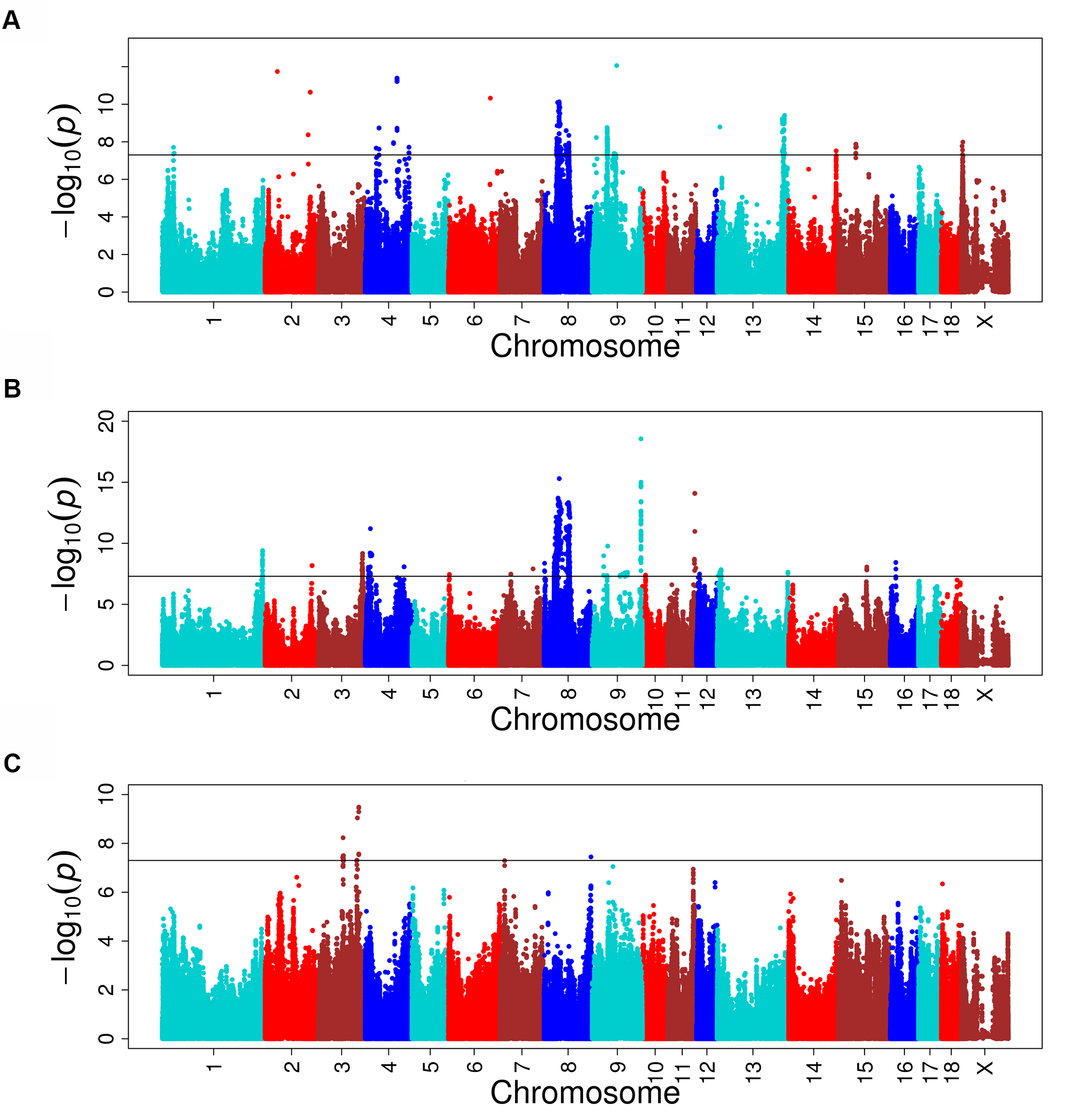


## **Supplementary Figure S9. Multi-trait GWAS results.** The tested multiple traits include GRAN, GRAR, MCH and MCHC at stage of day-46 (a); include HGB, HCT, RBC, GRAN, GRAR, MON and MONR at stage of day-240 (b); include RDW, PCT, PLT, LYM and LYMA at stage of day-240 (c). The y-axis and x-axis represent the negative log10 P value of the SNPs and the genomic positions separated by chromosomes, respectively, and the black solid lines indicate the significance threshold (negative log10 5E-08).


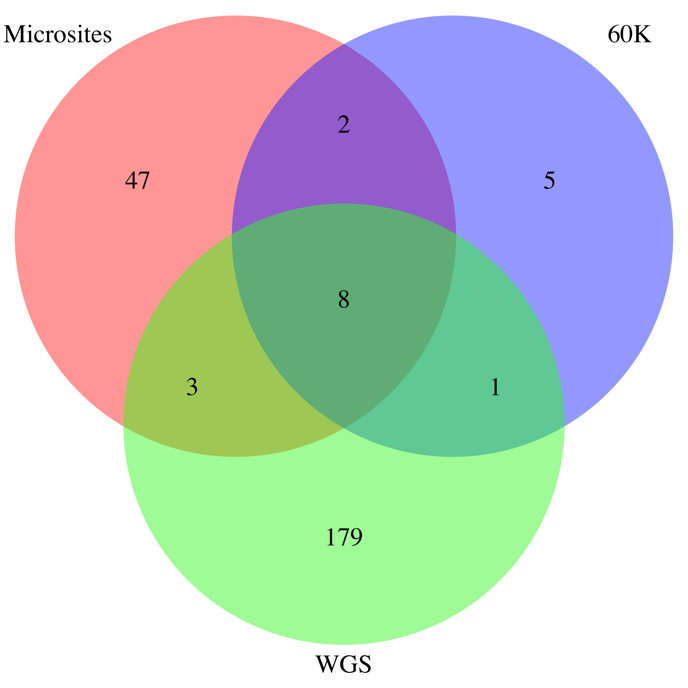


Supplementary Figure S10. **The concordance and difference in the identification of QTLs in three different studies with same phenotypic data.** Microsites, 60K and WGS indicate that QTLs were identified by QTL mapping, 60K GWAS and whole-genome GWAS, respectively.

## Supplementary Tables

**Supplementary Table S1**

**Table S1. The number for traits**

| **Order** | **Trait** | **Number** |
| --- | --- | --- |
| 1 | HCT18 | 778 |
| 2 | HGB18 | 779 |
| 3 | MCH18 | 778 |
| 4 | MCHC18 | 777 |
| 5 | MCV18 | 777 |
| 6 | RBC18 | 778 |
| 7 | RDW18 | 758 |
| 8 | HCT46 | 781 |
| 9 | HGB46 | 780 |
| 10 | MCH46 | 779 |
| 11 | MCHC46 | 767 |
| 12 | MCV46 | 781 |
| 13 | RBC46 | 781 |
| 14 | RDW46 | 735 |
| 15 | HCT240 | 910 |
| 16 | HGB240 | 924 |
| 17 | MCH240 | 909 |
| 18 | MCHC240 | 909 |
| 19 | MCV240 | 918 |
| 20 | RBC240 | 910 |
| 21 | RDW240 | 906 |
| 22 | GRAN18 | 777 |
| 23 | GRAR18 | 777 |
| 24 | LYM18 | 777 |
| 25 | LYMA18 | 780 |
| 26 | MON18 | 181 |
| 27 | MONR18 | 181 |
| **Order** | **Trait** | **Number** |
| 28 | WBC18 | 780 |
| 29 | GRAN46 | 772 |
| 30 | GRAR46 | 776 |
| 31 | LYM46 | 777 |
| 32 | LYMA46 | 781 |
| 33 | MON46 | 189 |
| 34 | MONR46 | 193 |
| 35 | WBC46 | 782 |
| 36 | GRAN240 | 712 |
| 37 | GRAR240 | 814 |
| 38 | LYM240 | 923 |
| 39 | LYMA240 | 924 |
| 40 | MON240 | 662 |
| 41 | MONR240 | 662 |
| 42 | WBC240 | 923 |
| 43 | MPV18 | 250 |
| 44 | PCT18 | 181 |
| 45 | PDW18 | 250 |
| 46 | PLT18 | 738 |
| 47 | MPV46 | 206 |
| 48 | PCT46 | 205 |
| 49 | PDW46 | 211 |
| 50 | PLT46 | 708 |
| 51 | MPV240 | 666 |
| 52 | PCT240 | 661 |
| 53 | PDW240 | 667 |
| 54 | PLT240 | 909 |

### Supplementary Table S2

**Table S2. Genome-wide significant loci associated with hematological traits at three different stage (exclude the erythroid traits at stage of day-18).**

| **Trait ^1^** | **Chr ^2^** | **Pos (bp) ^3^** | **P-value** | **Num ^4^** | **Nearest gene ^5^** | **Dis (bp) ^6^** | **Maf ^7^** |
| --- | --- | --- | --- | --- | --- | --- | --- |
| HCT46 | 2 | 37,589,362 | 3.23E-14 | 1 | *LUZP2* | within | 0.482 |
| HCT46 | 2 | 138,505,314 | 4.63E-14 | 8 | *HINT1* | 195,578 | 0.015 |
| HCT46 | 4 | 42,387,493 | 5.02E-14 | 996 | *MTDH* | 221,046 | 0.012 |
| HCT46 | 4 | 134,127,019 | 1.89E-09 | 10 | *ALG14* | within | 0.012 |
| HCT46 | 6 | 150,157,252 | 1.08E-11 | 3 | *NA* | NA | 0.013 |
| HCT46 | 7 | 48,391,303 | 1.46E-08 | 1 | *ADGRF5* | within | 0.012 |
| HCT46 | 9 | 74,677,603 | 2.93E-15 | 1 | *CD46* | 7,342 | 0.483 |
| HCT46 | 9 | 12,388,970 | 9.21E-09 | 1 | *CAPN5* | 47,885 | 0.011 |
| HCT46 | 10 | 64,996,011 | 1.55E-11 | 8 | *CAMK1D* | 25,560 | 0.024 |
| HCT46 | 11 | 39,480,779 | 3.04E-11 | 3 | *NA* | NA | 0.493 |
| HCT46 | 11 | 72,177,151 | 4.61E-09 | 5 | *DNAJC3* | 15,372 | 0.013 |
| HCT46 | 13 | 8,161,754 | 2.93E-12 | 1 | *SGOL1* | 55,210 | 0.012 |
| HCT46 | 13 | 204,316,849 | 2.23E-11 | 841 | *KRTAP27-1* | 24,745 | 0.011 |
| HCT46 | 14 | 142,211,660 | 8.12E-09 | 2 | *PLPP4* | 228,504 | 0.015 |
| HCT46 | 15 | 121,228,442 | 3.24E-10 | 5 | *ZDBF2* | 90,572 | 0.012 |
| HCT46 | 15 | 53,826,518 | 5.41E-10 | 89 | *FAM149A* | 2,910 | 0.012 |
| HCT46 | 16 | 71,861,753 | 5.99E-09 | 1 | *ITK* | 45,415 | 0.012 |
| HCT46 | 17 | 11,326,271 | 3.55E-11 | 271 | *ZMAT4* | 19,926 | 0.012 |
| HCT46 | X | 2,560,202 | 1.38E-10 | 1 | *NLGN4X* | 206,569 | 0.025 |
| MCH46 | 1 | 163,409,646 | 1.46E-08 | 1 | *GALR1* | 416,071 | 0.014 |
| MCH46 | 1 | 303,912,469 | 3.94E-08 | 1 | *USP20* | within | 0.015 |
| MCH46 | 4 | 8,547,762 | 7.93E-16 | 8 | *LRRC6* | 22,952 | 0.011 |
| MCH46 | 6 | 145,722,245 | 8.55E-12 | 17 | *TTC4* | 7,050 | 0.015 |
| MCH46 | 8 | 6,777,232 | 2.65E-09 | 78 | *NA* | NA | 0.01 |
| MCH46 | 10 | 71,495,904 | 9.18E-09 | 130 | *NET1* | 47,567 | 0.018 |
| MCH46 | 12 | 57,775,049 | 9.1E-12 | 38 | *MYH1* | 192,112 | 0.027 |
| MCH46 | 17 | 62,987,398 | 2.39E-08 | 7 | *DOK5* | 439,348 | 0.014 |
| MCHC46 | 2 | 37,589,362 | 4.03E-15 | 1 | *LUZP2* | within | 0.482 |
| MCHC46 | 2 | 138,505,314 | 7.69E-13 | 8 | *HINT1* | 195,578 | 0.016 |
| MCHC46 | 4 | 97,403,620 | 1.45E-10 | 88 | *ARHGAP30* | 14,036 | 0.01 |
| MCHC46 | 4 | 42,387,493 | 2.67E-10 | 336 | *MTDH* | 221,046 | 0.012 |
| MCHC46 | 6 | 150,157,252 | 2.52E-08 | 3 | *NA* | NA | 0.013 |
| MCHC46 | 9 | 74,677,603 | 2.32E-15 | 1 | *CD46* | 7,342 | 0.482 |
| MCHC46 | 9 | 12,388,970 | 1.37E-09 | 1 | *CAPN5* | 47,885 | 0.01 |
| MCHC46 | 10 | 64,996,148 | 3.73E-08 | 3 | *CAMK1D* | 25,697 | 0.025 |
| MCHC46 | 11 | 39,480,779 | 3.96E-08 | 3 | *NA* | NA | 0.493 |
| MCHC46 | 13 | 205,009,763 | 1.42E-10 | 1093 | *TIAM1* | 162,646 | 0.011 |
| MCHC46 | 13 | 8,161,754 | 1.75E-08 | 1 | *SGOL1* | 55,210 | 0.012 |
| MCHC46 | 14 | 61,461,447 | 1.35E-08 | 1 | *KCNK1* | within | 0.017 |
| MCHC46 | 15 | 52,217,467 | 5.97E-11 | 89 | *IRF2* | within | 0.012 |
| MCHC46 | 17 | 1,678,051 | 1.1E-08 | 19 | *C8orf48* | 268,619 | 0.021 |
| MCHC46 | X | 2,560,202 | 5.49E-10 | 1 | *NLGN4X* | 206,569 | 0.026 |
| MCV46 | 1 | 246,765,696 | 2.78E-08 | 109 | *DMRT2* | 177,876 | 0.018 |
| MCV46 | 2 | 37,589,362 | 4.49E-17 | 1 | *LUZP2* | within | 0.482 |
| MCV46 | 2 | 138,505,314 | 2.86E-13 | 8 | *HINT1* | 195,578 | 0.015 |
| MCV46 | 3 | 11,287,310 | 1.46E-09 | 17 | *GTF2IRD1* | within | 0.014 |
| MCV46 | 4 | 134,127,019 | 1.01E-19 | 1414 | *ALG14* | within | 0.012 |
| MCV46 | 4 | 42,387,493 | 4.51E-18 | 399 | *MTDH* | 42,387,493 | 0.012 |
| MCV46 | 6 | 150,157,252 | 4.44E-26 | 66 | *NA* | NA | 0.013 |
| MCV46 | 8 | 124,476,946 | 1.2E-08 | 4 | *INTS12* | 53,644 | 0.011 |
| MCV46 | 8 | 81,955,434 | 3.95E-08 | 1 | *PRSS48* | 465,295 | 0.226 |
| MCV46 | 9 | 74,677,603 | 2.4E-17 | 4 | *CD46* | 7,342 | 0.483 |
| MCV46 | 9 | 12,388,970 | 1.79E-16 | 1 | *CAPN5* | 47,885 | 0.011 |
| MCV46 | 10 | 64,996,011 | 9.88E-12 | 8 | *CAMK1D* | 25,560 | 0.024 |
| MCV46 | 10 | 18,901,148 | 3.86E-10 | 2 | *ZBTB18* | 454,854 | 0.01 |
| MCV46 | 11 | 39,480,779 | 1.96E-15 | 3 | *NA* | NA | 0.493 |
| MCV46 | 11 | 72,177,232 | 3.31E-13 | 6 | *DNAJC3* | 15,453 | 0.015 |
| MCV46 | 13 | 8,161,754 | 6.67E-19 | 5 | *SGOL1* | 55,210 | 0.012 |
| MCV46 | 13 | 200,367,851 | 2.01E-18 | 2576 | *ADAMTS1* | 35,741 | 0.011 |
| MCV46 | 13 | 22,344,910 | 1.03E-10 | 14 | *ARPP21* | 410,660 | 0.022 |
| MCV46 | 13 | 121,228,442 | 7.05E-09 | 1 | *NLGN1* | 289,784 | 0.012 |
| MCV46 | 14 | 78,622,809 | 6.16E-15 | 4987 | *HK1* | 101,510 | 0.013 |
| MCV46 | 15 | 53,826,518 | 3.67E-12 | 105 | *FAM149A* | 2,910 | 0.012 |
| MCV46 | 17 | 3,317,949 | 4.93E-15 | 778 | *TUSC3* | 89,296 | 0.012 |
| MCV46 | X | 2,560,202 | 7.84E-16 | 54 | *NLGN4X* | 206,569 | 0.025 |
| RDW46 | 1 | 304,664,479 | 1.8E-08 | 1 | *PRDM12* | 2,365 | 0.068 |
| RDW46 | 8 | 5,733,734 | 4.68E-08 | 1 | *OTOP1* | within | 0.017 |
| RDW46 | 9 | 2,023,201 | 4.05E-10 | 14 | *OVCH2* | 284,752 | 0.073 |
| HCT240 | 7 | 45,401,990 | 1.97E-10 | 863 | *CDC5L* | 49,879 | 0.191 |
| HCT240 | 7 | 34,839,473 | 2.00E-09 | 585 | *GRM4* | within | 0.46 |
| HCT240 | 7 | 53,773,298 | 1.34E-09 | 162 | *RASGRF1* | within | 0.269 |
| HGB240 | 7 | 34,839,473 | 3.76E-10 | 708 | *GRM4* | within | 0.458 |
| HGB240 | 7 | 45,095,721 | 1.01E-08 | 45 | *SLC29A1* | within | 0.186 |
| HGB240 | 7 | 53,773,298 | 1.98E-08 | 5 | *RASGRF1* | within | 0.266 |
| MCH240 | 8 | 42,121,169 | 1.09E-15 | 9562 | *LNX1* | 229,929 | 0.347 |
| MCH240 | 8 | 76,300,036 | 9.66E-13 | 4251 | *SHROOM3* | 147,226 | 0.341 |
| MCH240 | 11 | 77,754,820 | 1.97E-09 | 23 | *FGF14* | within | 0.212 |
| MCHC240 | 6 | 7,889,499 | 1.14E-08 | 62 | *CDYL2* | 193,738 | 0.017 |
| MCHC240 | 17 | 17,100,124 | 1.52E-09 | 248 | *FERMT1* | 596,148 | 0.013 |
| MCV240 | 8 | 46,494,630 | 2.9E-15 | 12785 | *GUCY1A3* | within | 0.357 |
| MCV240 | 8 | 74,880,093 | 1.43E-12 | 6165 | *PARM1* | 127,742 | 0.263 |
| MCV240 | X | 48,628,584 | 3.59E-09 | 328 | *CLCN5* | 77,482 | 0.213 |
| MCV240 | X | 61,999,126 | 6.20E-09 | 237 | *STARD8* | within | 0.215 |
| RBC240 | 8 | 42,121,169 | 6.14E-10 | 506 | *LNX1* | 229,929 | 0.347 |
| RDW240 | X | 10,362,664 | 1.11E-11 | 97 | *PRPS2* | 8,621 | 0.415 |
| RDW240 | X | 103,239,211 | 1.99E-08 | 2 | *COL4A6* | 103,174 | 0.283 |
| GRAN18 | 3 | 2,968,881 | 1.32E-08 | 7 | *CHST12* | 682,305 | 0.011 |
| GRAN18 | 5 | 62,940,830 | 3.09E-09 | 240 | *ETV6* | 138,286 | 0.022 |
| GRAN18 | 5 | 9,651,960 | 1.84E-08 | 1 | *APOL6* | 1,380 | 0.014 |
| GRAN18 | 7 | 129,191,220 | 8.65E-10 | 9 | *RCOR1* | within | 0.013 |
| GRAN18 | 11 | 79,825,025 | 4.89E-08 | 1 | *NA* | NA | 0.021 |
| GRAR18 | 8 | 10,485,550 | 4.38E-09 | 35 | *CC2D2A* | within | 0.055 |
| LYM18 | 1 | 7,535,697 | 3.99E-09 | 2 | *NA* | NA | 0.012 |
| LYM18 | 5 | 33,535,414 | 4.64E-08 | 2 | *LLPH* | 183,969 | 0.058 |
| LYM18 | 7 | 102,771,321 | 6.19E-11 | 2 | *DNAL1* | within | 0.019 |
| LYM18 | 11 | 79,807,837 | 2.13E-10 | 82 | *NA* | NA | 0.017 |
| LYM18 | 16 | 83,915,502 | 4.93E-10 | 20 | *NA* | NA | 0.014 |
| LYM18 | 17 | 50,736,762 | 2.03E-10 | 522 | *PTPRT* | 22,728 | 0.08 |
| LYMA18 | 17 | 68,931,360 | 2E-08 | 8 | *CDH4* | within | 0.022 |
| MON18 | 1 | 115,872,507 | 1.96E-09 | 10 | *NA* | NA | 0.014 |
| MON18 | 1 | 290,048,081 | 1.04E-08 | 8 | *TLR4* | 262,234 | 0.019 |
| MON18 | 2 | 144,397,384 | 2.21E-08 | 62 | *SMAD5* | 342,652 | 0.014 |
| MON18 | 4 | 6,276,295 | 1.28E-08 | 11 | *CU151851.1* | 38,414 | 0.017 |
| MON18 | 5 | 66,039,834 | 8.2E-10 | 34 | *RBP5* | within | 0.022 |
| MON18 | 7 | 24,463,360 | 2.04E-12 | 1176 | *TRIM27* | 11,747 | 0.025 |
| MON18 | 11 | 73,189,920 | 7.51E-10 | 77 | *HS6ST3* | 2,271 | 0.036 |
| MON18 | 14 | 13,181,606 | 1.87E-10 | 18 | *PNOC* | 75,381 | 0.466 |
| MON18 | 17 | 65,318,413 | 1.21E-09 | 31 | *PMEPA1* | 104,066 | 0.036 |
| MON18 | X | 36,744,806 | 5.79E-09 | 26 | *PRRG1* | within | 0.025 |
| MON18 | X | 110,538,300 | 1.05E-08 | 20 | *SLC6A14* | 398,556 | 0.034 |
| MONR18 | 1 | 144,997,139 | 1.8E-09 | 219 | *RTF1* | within | 0.041 |
| MONR18 | 1 | 290,048,081 | 1.41E-08 | 8 | *TLR4* | 262,234 | 0.019 |
| MONR18 | 2 | 1,469,464 | 1.23E-12 | 5 | *FADD* | 79,374 | 0.011 |
| MONR18 | 3 | 120,842,827 | 4.05E-09 | 97 | *EFR3B* | 43,275 | 0.025 |
| MONR18 | 4 | 6,276,295 | 4.57E-11 | 109 | *CU151851.1* | 38,414 | 0.017 |
| MONR18 | 4 | 97,417,040 | 1.07E-08 | 8 | *ARHGAP30* | 27,456 | 0.112 |
| MONR18 | 4 | 124,466,585 | 3.26E-10 | 1 | *NA* | NA | 0.031 |
| MONR18 | 5 | 66,039,834 | 1.38E-13 | 39 | *RBP5* | within | 0.022 |
| MONR18 | 7 | 24,458,274 | 2.7E-11 | 269 | *TRIM27* | 6,661 | 0.025 |
| MONR18 | 9 | 9,096,104 | 4.05E-08 | 46 | *PAAF1* | within | 0.037 |
| MONR18 | 11 | 73,190,604 | 1.25E-13 | 733 | *HS6ST3* | 1,587 | 0.014 |
| MONR18 | 15 | 32,297,514 | 7.78E-11 | 9 | *NA* | NA | 0.011 |
| MONR18 | X | 110,538,300 | 2.44E-15 | 247 | *SLC6A14* | 398,556 | 0.034 |
| WBC18 | 1 | 7,535,697 | 8.37E-09 | 2 | *NA* | NA | 0.012 |
| WBC18 | 3 | 2,970,216 | 2.59E-10 | 7 | *CHST12* | 683,640 | 0.01 |
| WBC18 | 5 | 16,587,239 | 1.38E-08 | 5 | *FAM186A* | 40,488 | 0.013 |
| WBC18 | 5 | 33,535,414 | 1.86E-08 | 3 | *LLPH* | 183,969 | 0.058 |
| WBC18 | 7 | 102,771,321 | 3.9E-11 | 2 | *DNAL1* | within | 0.019 |
| WBC18 | 11 | 79,807,837 | 6.46E-13 | 113 | *NA* | NA | 0.017 |
| WBC18 | 13 | 15,986,713 | 8E-10 | 4 | *SLC4A7* | 436,645 | 0.015 |
| WBC18 | 15 | 137,359,457 | 4.56E-10 | 4 | *PAX3* | 259,321 | 0.01 |
| WBC18 | 16 | 83,915,502 | 2.92E-11 | 20 | *NA* | NA | 0.014 |
| WBC18 | 17 | 50,736,762 | 7.1E-10 | 406 | *PTPRT* | 22,728 | 0.08 |
| GRAN46 | 1 | 34,669,477 | 1.14E-08 | 2 | *TAAR6* | 7,211 | 0.098 |
| GRAN46 | X | 5,114,932 | 1.8E-08 | 9 | *ANOS1* | 251,083 | 0.361 |
| GRAR46 | X | 5,368,317 | 2.26E-09 | 22 | *ANOS1* | within | 0.274 |
| LYMA46 | X | 5,434,292 | 4.92E-09 | 19 | *ANOS1* | within | 0.443 |
| MONR46 | 2 | 9,708,290 | 3.98E-10 | 1 | *TKFC* | within | 0.013 |
| MONR46 | 4 | 131,768,991 | 4.14E-08 | 4 | *NA* | NA | 0.036 |
| MONR46 | 7 | 27,718,253 | 3.82E-08 | 71 | *TNXB* | within | 0.034 |
| MONR46 | 8 | 5,147,229 | 4.3E-08 | 8 | *MSX1* | within | 0.018 |
| MONR46 | 9 | 46,668,542 | 1.29E-08 | 3 | *NNMT* | 37,528 | 0.01 |
| MONR46 | 15 | 28,493,387 | 2.35E-08 | 11 | *C2orf76* | 24,577 | 0.023 |
| MONR46 | 17 | 66,496,954 | 5.89E-09 | 2 | *NELFCD* | 2,529 | 0.01 |
| GRAN240 | 3 | 134,101,283 | 1.92E-08 | 1 | *E2F6* | 17,394 | 0.278 |
| GRAN240 | X | 10,362,676 | 1.57E-09 | 12 | *PRPS2* | 8,609 | 0.434 |
| GRAR240 | 17 | 20,750,926 | 2.54E-08 | 204 | *PLCB4* | 19,123 | 0.012 |
| GRAR240 | X | 10,362,676 | 4.39E-10 | 19 | *PRPS2* | 8,609 | 0.449 |
| MON240 | 4 | 15,955,118 | 3.16E-13 | 859 | *TATDN1* | 139,067 | 0.011 |
| MON240 | 7 | 128,281,109 | 4.89E-08 | 1 | *CYP46A1* | within | 0.024 |
| MON240 | 9 | 149,015,790 | 8.9E-21 | 1059 | *VWC2* | 493,517 | 0.016 |
| MON240 | 9 | 35,536,888 | 1.36E-12 | 47 | *CNTN5* | 9,933 | 0.014 |
| MON240 | 11 | 82,484,801 | 4.31E-08 | 12 | *FAM155A* | 258,580 | 0.02 |
| MON240 | 13 | 16,916,993 | 2.58E-08 | 8 | *AZI2* | 381,837 | 0.014 |
| MON240 | 16 | 19,442,052 | 8.47E-10 | 26 | *PDZD2* | 294,881 | 0.011 |
| MONR240 | 4 | 15,955,118 | 1.81E-12 | 106 | *TATDN1* | 139,067 | 0.011 |
| MONR240 | 9 | 149,015,790 | 3.8E-18 | 193 | *VWC2* | 493,517 | 0.016 |
| MONR240 | 9 | 35,536,888 | 6.48E-09 | 38 | *CNTN5* | 9,933 | 0.014 |
| MONR240 | 10 | 736,951 | 6.62E-09 | 11 | *COG7* | 482,872 | 0.046 |
| MONR240 | 11 | 82,484,801 | 9.68E-13 | 16 | *FAM155A* | 258,580 | 0.02 |
| MONR240 | 12 | 54,110,317 | 2.44E-09 | 1 | *MINK1* | 7,895 | 0.01 |
| MONR240 | 13 | 16,916,993 | 1.11E-09 | 8 | *AZI2* | 381,837 | 0.014 |
| MPV18 | 16 | 2,799,106 | 4.44E-10 | 512 | *NA* | NA | 0.026 |
| PDW18 | 5 | 61,481,315 | 2.2E-08 | 13 | *ATF7IP* | within | 0.024 |
| PDW18 | 6 | 4,342,979 | 4.48E-08 | 1 | *USP10* | -48,985 | 0.156 |
| PDW18 | 8 | 66,808,851 | 7.33E-10 | 46 | *TECRL* | 478,869 | 0.43 |
| PDW18 | X | 9,102,358 | 5.19E-10 | 5 | *MSL3* | 190,278 | 0.06 |
| PDW18 | X | 13,909,268 | 3.90E-08 | 1 | *CTPS2* | 86,958 | 0.049 |
| MPV46 | 9 | 152,123,000 | 2.6E-09 | 1 | *NA* | NA | 0.024 |
| PCT46 | 3 | 6,046,306 | 2.51E-08 | 1 | *TRRAP* | within | 0.025 |
| PCT46 | 6 | 150,766,642 | 1.32E-08 | 1 | *SPATA6* | 43,344 | 0.056 |
| PCT46 | 16 | 6,619,649 | 1.81E-11 | 2 | *MYO10* | 126,967 | 0.096 |
| PCT46 | X | 2,560,202 | 2.71E-08 | 1 | *NLGN4X* | 206,569 | 0.039 |
| PDW46 | 11 | 77,441,619 | 1.19E-09 | 14 | *ITGBL1* | within | 0.239 |
| PLT46 | 5 | 95,687,215 | 1E-10 | 536 | *DCN* | 422,451 | 0.056 |

1 Abbreviations of hematological traits, i.e. HCT18 is hematocrit at stage of day-18.

2 Chromosomal locations of the most significant SNPs.

3 Positions of the most significant SNPs according to *sus scrofa* 10.2 genome assembly.

4 The number of SNPs reached the threshold (*P* < 5E-08).

5 The nearest annotated genes from the most significant SNPs.

6 The distance from the most significant SNPs to the nearest genes.

7 the frequency of the most significant SNPs.

### Supplementary Table S3

**Table S3. Genome-wide significant loci associated with hematological traits at different stages by multi-trait GWAS.**

| **Trait ^1^** | **Chr ^2^** | **Pos (bp) ^3^** | **P-value** | **Nearest gene ^4^** | **Dis (bp) ^5^** | **Maf ^6^** | **Beta1** | **Beta2** | **Beta3** | **Beta4** | **Beta5** | **Beta6** | **Beta7** |
| --- | --- | --- | --- | --- | --- | --- | --- | --- | --- | --- | --- | --- | --- |
| MT-GWAS1 | 3 | 27,061,999 | 6.094E-10 | *TMC7* | 8,270 | 0.011 | 0.05 | -0.11 | -0.44 | 8.47 | - | - | - |
| MT-GWAS1 | 3 | 115,277,228 | 2.327E-08 | *LCLAT1* | 94,305 | 0.01 | 0.06 | 5.44 | 0.70 | 9.80 | - | - | - |
| MT-GWAS1 | 5 | 63,702,557 | 5.02E-09 | *BCL2L14* | within | 0.014 | 0.04 | -1.32 | -1.07 | 5.64 | - | - | - |
| MT-GWAS1 | 7 | 15,253,872 | 8.067E-11 | *RNF144B* | 393,298 | 0.01 | 0.05 | 1.76 | 0.18 | 8.57 | - | - | - |
| MT-GWAS1 | 7 | 116,728,782 | 3.028E-08 | *KCNK10* | 32,979 | 0.327 | -0.02 | -7.87 | -0.62 | -1.71 | - | - | - |
| MT-GWAS1 | 9 | 144,340,814 | 3.218E-09 | *DTL* | 17,902 | 0.034 | -0.02 | -9.87 | -0.55 | -0.85 | - | - | - |
| MT-GWAS1 | 10 | 1,107,823 | 2.353E-09 | *NA* | NA | 0.01 | -0.08 | -20.51 | -1.76 | -9.93 | - | - | - |
| MT-GWAS1 | 10 | 23,888,810 | 1.429E-11 | *KCNT2* | 7,750 | 0.01 | -0.09 | -23.80 | -1.64 | -9.49 | - | - | - |
| MT-GWAS2 | 14 | 145,780,044 | 3.034E-08 | *ZRANB1* | 25,027 | 0.095 | 0.54 | 21.78 | 0.83 | 5.94 | - | - | - |
| MT-GWAS3 | 1 | 307,696,657 | 3.905E-10 | *BRD3* | 189,227 | 0.053 | -0.01 | -4.16 | -0.08 | 0.92 | -0.30 | 0.14 | 1.04 |
| MT-GWAS3 | 2 | 143,621,656 | 6.702E-09 | *SLC25A48* | within | 0.013 | -0.01 | -0.69 | -0.23 | -2.28 | -4.75 | 0.21 | 2.13 |
| MT-GWAS3 | 6 | 2,975,322 | 3.525E-08 | *MTHFSD* | 188,918 | 0.028 | -0.01 | -1.42 | -0.28 | -1.55 | 7.17 | -0.12 | -0.07 |
| MT-GWAS3 | 15 | 85,801,819 | 8.695E-09 | *UBR3* | 422,452 | 0.422 | -0.01 | -3.37 | -0.12 | -1.52 | -4.86 | 0.02 | 0.19 |
| MT-GWAS4 | 3 | 76,663,552 | 5.879E-09 | *ANTXR1* | within | 0.033 | -0.12 | 3.36 | 11.74 | 0.02 | 56.84 | - | - |
| MT-GWAS4 | 3 | 124,826,140 | 3.271E-10 | *NA* | NA | 0.01 | -0.21 | -0.06 | -0.25 | 0.10 | 17.39 | - | - |
| MT-GWAS4 | 8 | 144,180,997 | 3.58E-08 | *GPAT3* | 29,510 | 0.012 | 1.93 | -0.55 | -5.30 | 0.07 | 47.17 | - | - |

MT-GWAS1 includes HCT, HGB, MCH and MCV at stage of day-18; MT-GWAS2 include GRAN, GRAR, MCH and MCHC at stage of day-46; MT-GWAS3 includes HGB, HCT, RBC, GRAN, GRAR, MON and MONR at stage of day-240; MT-GWAS4 includes RDW, PCT, PLT, LYM and LYMA at stage of day-240.

1 Abbreviations of hematological traits, i.e. HCT18 is hematocrit at stage of day-18.

2 Chromosomal locations of the most significant SNPs.

3 Positions of the most significant SNPs according to *sus scrofa* 10.2 genome assembly.

4 The nearest annotated genes from the most significant SNPs.

5 The distance from the most significant SNPs to the nearest genes.

6 the frequency of the most significant SNPs.

Beta1, Beta2, Beta3, Beta4, Beta5, Beta6 and Beta7 are the estimated SNP effect size.

**Supplementary Table S4**

**Table S4. The concordance in three different studies with same phenotypic data.**

| **Order** | **Chr** | **Trait** | **Position(cM/Mb)^1^** | **Number of QTLs^2^** | | |
| --- | --- | --- | --- | --- | --- | --- |
|  |  |  |  | **Microsites** | **60K** | **WGS** |
| 1 | 4 | HCT46 | 65 (32.0–84.0) | 1 | 0 | 1 |
| 2 | 6 | MCV240 | 99 (21.0–188.0) | 1 | 0 | 1 |
| 3 | 7 | HCT240 | 57 (49.0–70.0) | 1 | 3 | 3 |
| 4 | 7 | HGB240 | 57 (57.0–67.0) | 1 | 3 | 3 |
| 5 | 8 | MCH18 | 74 (73.0–77.0) | 1 | 2 | 0 |
| 6 | 8 | MCH46 | 74 (73.0–89.0) | 1 | 0 | 1 |
| 7 | 8 | MCH240 | 74 (70.0–76.0) | 1 | 1 | 1 |
| 8 | 8 | MCV18 | 77 (74.0–77.0) | 1 | 2 | 1 |
| 9 | 8 | MCV46 | 75 (72.0–140.0) | 1 | 2 | 2 |
| 10 | 8 | MCV240 | 74 (69.0–76.0) | 1 | 1 | 1 |
| 11 | 8 | RBC18 | 73 (47.0–86.5) | 1 | 2 | 2 |
| 12 | 8 | RBC240 | 75 (65.0–77.0) | 1 | 2 | 1 |
| 13 | 7 | WBC240 | 60 (53.0–137.0) | 1 | 1 | 0 |
| 14 | 4 | MCHC46 | 42,489,363 | 0 | 1 | 1 |

^1^ QTL positions in centimorgan (cM) and megabase (Mb).

^2^ QTL numbers identified by QTL mapping, 60K GWAS and whole-genome GWAS using the same F_2_ population.
